# The synergistic compatibility mechanisms of fuzi against chronic heart failure in animals: A systematic review and meta-analysis

**DOI:** 10.3389/fphar.2022.954253

**Published:** 2022-09-14

**Authors:** Xingyu Liu, Xiaofang Xie, Maozhu Luo, Yuting Zhao, Mengting Li, Fu Peng, Cheng Peng

**Affiliations:** ^1^ State Key Laboratory of Southwestern Chinese Medicine Resources, Chengdu University of Traditional Chinese Medicine, Chengdu, China; ^2^ Personalized Drug Therapy Key Laboratory of Sichuan Province, Department of Pharmacy, Sichuan Provincial People’s Hospital, School of Medicine, University of Electronic Science and Technology of China, Chengdu, Sichuan, China; ^3^ West China School of Pharmacy, Sichuan University, Chengdu, China

**Keywords:** aconiti lateralis radix praeparata, chronic heart failure, traditional Chinese medicine, synergistic compatibility, mechanism, meta-analysis

## Abstract

**Background:** Fuzi’s compatibilities with other medicines are effective treatments for chronic heart failure. Pre-clinical animal experiments have indicated many possible synergistic compatibility mechanisms of it, but the results were not reliable and reproducible enough. Therefore, we performed this systematic review and meta-analysis of pre-clinical animal studies to integrate evidence, conducted both qualitative and quantitative evaluations of the compatibility and summarized potential synergistic mechanisms.

**Method:** An exhaustive search was conducted for potentially relevant studies in nine online databases. The selection criteria were based on the Participants, Interventions, Control, Outcomes, and Study designs strategy. The SYRCLE risk of bias tool for animal trials was used to perform the methodological quality assessment. RevMan V.5.3 and STATA/SE 15.1 were used to perform the meta-analysis following the Cochrane Handbook for Systematic Reviews of Interventions.

**Result:** 24 studies were included in the systematic review and meta-analysis. 12 outcomes were evaluated in the meta-analysis, including BNP, HR, HWI, ALD, LVEDP, LVSP, EF, FS, +dP/dt_max_, −dP/dt_max_, TNF-α and the activity of Na ^+^ -K ^+^ -ATPase. Subgroup analyses were performed depending on the modeling methods and duration.

**Conclusion:** The synergistic Fuzi compatibility therapeutic effects against CHF animals were superior to those of Fuzi alone, as shown by improvements in cardiac function, resistance to ventricular remodeling and cardiac damage, regulation of myocardial energy metabolism disorder and RAAS, alleviation of inflammation, the metabolic process *in vivo*, and inhibition of cardiomyocyte apoptosis. Variations in CHF modeling methods and medication duration brought out possible model–effect and time-effect relationships.

## 1 Introduction

Heart failure (HF) is a global condition that affects approximately 38 million patients worldwide with increasing prevalence and incidence as a population ages (Braunwald, 2015). HF is not caused by a single factor but by a complex terminal-stage syndrome induced by various cardiovascular diseases and characterized by typical symptoms (e.g., breathlessness, ankle swelling, and fatigue) that may be accompanied by signs (e.g., elevated jugular venous pressure, pulmonary crackles, and peripheral edema) caused by a structural or functional cardiac abnormality, resulting in reduced cardiac output and elevated intracardiac pressures (Ponikowski et al., 2016). Depending on the time of onset, it is classified as acute and chronic HF (Pagliaro et al., 2020). Chronic heart failure (CHF) is one of the most common chronic conditions worldwide (Ewen et al., 2016), with an estimated prevalence of 26 million patients worldwide (Ponikowski et al., 2014). Pathogenic causes of CHF mainly include single or combined factors, such as reduced left ventricular myocardial function or dysfunction of the pericardium, endocardium, heart valves, great vessels, and the right ventricular myocardium. Related pathogenic mechanisms leading to CHF involve ventricular remodeling, increased hemodynamic overload, abnormal cardiac myocyte calcium circulation, ischemia-related dysfunction, excessive neuro-humoral stimulation, accelerated apoptosis, and genetic mutations (Dassanayaka and Jones, 2015). The health expenditures of CHF are considerable and will rise dramatically with an ageing population. Although significant advances and progress have been made in therapies and prevention, the 5-years mortality rate of CHF remains at 50%, which is the same as that of a malignant tumor (O'Gara et al., 2013), and the quality of life and prognosis remains poor (Savarese and Lund, 2017), which impose a huge burden on socioeconomics and medical resources (Lloyd-Jones et al., 2009). The development of more effective and promising treatment is of great importance and urgency.

In recent years, traditional Chinese medicine (TCM) has made great progress in treating CHF and plays a dominant role in numerous treatments, showing superior and unique efficacy in controlling symptoms, enhancing therapeutic effects, and improving prognosis, which has made TCM a popular treatment of CHF. According to the basic theory of Chinese medicine, CHF belongs to the categories of palpitation, chest discomfort, edema, and heart obstruction, and the causes and underlying mechanisms are defined as qi deficiency and yang deficiency, whose corresponding treatment is tonifying qi and warming yang. Statistically, the use of this treatment approach ranked first at 64.3% among all reported treatments of CHF (Xu, 2016).

Aconiti Lateralis Radix Praeparata (Fuzi in Chinese), the lateral root of *Aconitum carmichaelii* Debx., is a time-honored interior-warming TCM used in CHF that is usually processed into salty Fuzi, hei-shun-pian, or bai-fu-pian. The main bioactive components of Fuzi are diester–diterpene alkaloids (DDAs), which mainly include aconitine (AC), mesaconitine, and hypaconitine (HA). Fuzi is praised as one of the most effective TCMs for revitalizing yang and rescuing patients from collapse, indicating its pharmacological cardiotonic efficacy by elevating blood pressure, causing vasodilation, increasing blood flow volume, enhancing hypoxia tolerance and cold resistance, and providing anti-shock, anti-arrhythmic, and anti-myocardial ischemic activities. Powerful as Fuzi presents, attention should also be paid to its toxicity. Aconitine, the main bioactive part of Fuzi, serves as dual effects of toxicity and efficacy. For humans, the estimated lethal dose is 2 mg of pure aconitine, 5 ml of aconite tincture, and 1 g of the wild plant (Singh et al., 1986). In addition, it is especially known for its cardiotoxicity, with electrocardiograms presenting a transient decrease in heart rate, atrial and ventricular extrasystole, and tachycardia as well as non-paroxysmal ventricular tachycardia and ventricular fibrillation. Therefore, Fuzi is only used clinically after proper processing and always combined with other TCMs to reduce its toxic alkaloid content, thus enhancing the curative effects and alleviating adverse reactions.

The compatibility of TCMs is the fundamental principle of clinical Chinese medical administration, presenting the characteristics and edges of TCM. With proper compatibility, the therapeutic effects can be enhanced and the toxic and side effects decreased, widening clinical applications, regulating interactions, and sometimes resulting in new therapeutic effects. Compatibility refers to purposeful, organized, and well-founded combinations between TCMs based on the rule of *Qi Qing*. Literally, *Qi* means the number seven, and *Qing* represents emotions. Chinese ancient medical scholars summarized that there were seven kinds of compatibility relationships between TCMs: single, mutual reinforcement, mutual assistance, mutual restraint, mutual suppression, mutual inhibition, and antagonism. A TCM prescription is based on the compatibility of the ingredients. According to the contribution of each medicine to the final effect of a prescription, different roles are determined as sovereign, minister, assistant, and courier. Clinically, Fuzi is the most frequently used yang-warming medicine among TCMs at 80.3% (Chan, 2009), and it is most compatible with dried ginger (*Zingiber officinale* Rosc.) according to the rule of mutual reinforcement and is usually used in the treatment of yang collapse (e.g., Sini Decoction (SND)). The main bioactive component of dried ginger is gingerol, which is capable of resisting and reducing the toxicity of aconitine. Peng (2013) found that the compatibility of aconite and ginger can promote the dissolution of aconitine alkaloids, inhibit the transport of DDAs to the heart and their absorption in the body while promoting the absorption of monoester alkaloids, including benzoylaconine (BAC), benzoylhypaconine (BHA), and benzoylmesaconine (BMA), delaying their elimination *in vivo*. Ye et al. (2013) compared the content of curative alkaloids of dried ginger, fresh ginger, and sand-fried ginger after compatibility with Fuzi by referencing a high-performance liquid chromatography atlas and found that the content of curative alkaloids in the dried ginger’s compatibility group was higher than that of a simple Fuzi decoction, proving that dried ginger, when used with Fuzi, can increase the therapeutic effect. In addition, when Fuzi is combined with ginseng (*Panax ginseng* C.A. Mey.), mutual assistance makes it effective compatibility for both yang and qi collapse (e.g., Shenfu Decoction (SFD)). Pharmacology research (Cui et al., 2013) has found that ginsenoside, the main active ingredient of ginseng, can improve myocardial metabolism, protect the myocardial structure, and enhance cardiac contractility. Li et al. (2011) found that the water decoction of Fuzi in combination with ginseng at 1:2 enhanced the heart-strengthening effect by significantly improving the rats’ hemodynamic indexes relative to the effect of Fuzi water decoction alone. Another study (Li et al., 2013) found that the compatibility of Fuzi and ginseng also improved the myocardial diastolic and contraction function and changed the levels of N-terminal pro-brain natriuretic peptide, angiotensin II (Ang II), and atrial natriuretic peptide in the plasma of rats with acute heart failure, achieving the effects of alleviating HF.

In recent years, the clinical application of Fuzi against CHF is becoming more popular, along with which, however, are increasing aconite poisoning and inefficacious reports due to inappropriate processing, compatibility, or usage (Chan, 2011; Chen et al., 2012). Currently, prominent progress has been made in Fuzi’s chemical components, pharmacological effects, attenuated processing, and attenuated compatibility (Wei et al., 2019; Yang et al., 2019). However, a lack of further research on the mechanisms of synergistic compatibility of Fuzi against CHF remains. Although multiple possible mechanisms have been demonstrated through animal experiments, small sample size effects and individual differences still exist, making it hard to draw a specific and reliable conclusion. For a long time, quality requirements in clinical research are normally higher than in animal experiments, hence the derivative is a relatively comprehensive way of quality control and assessment of clinical interventions, resulting in, however, a poor human clinical and toxicological utility of animal experiments (Knight, 2007). A systematic review is conducive to assembling overall evidence, enlarging sample size, and lowering various risks of bias, thus systematic evaluations of high-qualified randomized controlled trials (RCTs) are acknowledged to be one the most reliable pieces of evidence when the efficacy of interventions needs proving. For the past few years, facing the fact that new drug test results on animals often failed to apply to clinical research (Van Norman, 2019; Van Norman, 2020), reminds people of the necessity of quality assessment of animal experiments. Ferreira et al. (Ferreira et al., 2020) addressed the importance of levelling the translational gap for the animal to human efficacy data and pointed out that selecting optimal animal models of disease may prevent the conducting of clinical trials, based on unreliable preclinical data. Without a doubt, a systematic review of animal experiments is of importance and beneficial to avoiding animal resource wasting, lowering experimental costs and preventing patients from unknown risks (van Meer et al., 2015).

All things considered, our study was designed to conduct a systematic review and meta-analysis of pre-clinical animal studies to integrate evidence from all relevant studies, conduct both qualitative and quantitative evaluations of the compatibility and summarize all potential synergistic mechanisms of compatibility, through which the reliability and accuracy can be increased. The purpose of this study was to 1) assess the efficacy of Fuzi compatibility with any other TCMs or their bioactive constituents against CHF by reviewing all related animal studies, 2) identify provable evidence for synergistic compatibility mechanisms of Fuzi for CHF, 3) evaluate the impact of possible publication bias and small-study effect, 4) explore the factors that may affect the efficacy of compatibility, and 5) hopefully serve as a reference for further clinical trials and applications.

## 2 Method

This study was performed following the Cochrane Handbook for Systematic Reviews of Interventions (Higgins et al., 2019). The protocol for this meta-analysis is available in PROSPERO (CRD42021290955).

### 2.1 Search strategy

An exhaustive search was conducted for potentially relevant studies in nine online databases: PubMed, the Cochrane Library, Web of Science, Embase, CINAHL Complete, China National Knowledge Infrastructure (CNKI), Wanfang Data Information Site, VIP Information Database, and China Biological Medicine Database (CBM). The language was restricted to Chinese and English, and the publication time ranged from the establishment of the databases to July 2022. The search strategy depended on MeSH terms with free words and was applied to English databases whereas the corresponding terms were used in Chinese databases. In addition, possibly related studies (e.g., conference literature) were also carefully identified through manual searching. The full retrieval details are attached in Supplementary Material, Supplementary Appendix S1.

### 2.2 Selection criteria

The selection criteria were based on the Participants, Interventions, Control, Outcomes, and Study designs (PICOS) strategy.

#### 2.2.1 Inclusion criteria

The following inclusion criteria were used:

1) Participants: rat or mice CHF models, with unrestricted modeling methods
2) Interventions: Fuzi or its bioactive constituents was administered with any other TCM or their bioactive constituents, with an unrestricted number of combined TCMs
3) Control: Fuzi or its constituents only were administered
4) Outcomes: brain natriuretic peptide (BNP), heart rate (HR), heart weight index (HWI), aldosterone (ALD), left ventricular end-diastolic pressure (LVEDP), left ventricular systolic pressure (LVSP), ejection fraction (EF), fractional shortening (FS), maximum rate of increase in left ventricular pressure (+dP/dt_max_), maximum rate of decrease in left ventricular pressure (−dP/dt_max_), Ang II, endothelin 1 (ET-1), tumor necrosis factor-α (TNF-α), the activity of Na^+^-K ^+^ -ATPase, creatine kinase (CK), lactate dehydrogenase (LDH) and adenosine triphosphate (ATP).
5) Study design: only randomized controlled trials (RCTs) in animals were analyzed.

#### 2.2.2 Exclusion criteria

1) Participants: models other than CHF, *in vitro* studies, or clinical trials in humans.
2) Interventions: administration combined with other non-TCM treatments (e.g., electroacupuncture) or without compatibility.
3) Control: administration combined with other TCM or treatments.
4) Study type: case reports, reviews, conference proceedings, catalogue, and technical achievement reports.
5) Duplicate publication.
6) Studies without full text.

### 2.3 Study screening

All retrieved articles were imported into EndNote 20, and duplicate studies were removed first. Then, two reviewers independently performed primary screening based on titles and abstracts, and studies inconsistent with the selection criteria were excluded directly. Next, a full-text screening for eligible articles was performed, and two reviewers recorded specific exclusion reasons independently. Finally, a cross-check of results between the two reviewers was performed to ensure the consistency of the screening. During this procedure, any disagreements between the two researchers were discussed with a third reviewer, and the final judgment was made.

### 2.4 Data extraction

A form for data collection was designed and established in Microsoft Excel 2021, and two reviewers extracted the following items from the included papers independently:

1) Study ID: first author’s name and publication year.
2) Participant’s information: number, sex, species, weight, and CHF model.
3) Administration: interventions and control measures.
4) Durations.
5) Outcome indexes mentioned in ***2.2.1***.

All outcome measures were analyzed as continuous variables, estimated mean and standard deviation values were extracted, and each variable was extracted from the last time point. For trials with more than one experimental group but sharing a common control group, the control group was divided into multiple groups with its number in accordance with experimental groups, and these added comparisons were also incorporated into the meta-analysis (Higgins et al., 2019). An email of the request was sent to authors of related studies for more information when the details of studies or data about outcomes were missing or only presented in graphs. If relevant data were not available or insufficient for quantitative analysis, qualitative analysis was performed.

### 2.5 Quality assessment

Two researchers conducted quality assessments independently, and the SYRCLE risk of bias tool for animal trials was adopted to perform the methodological quality assessment (Hooijmans et al., 2014), which consisted of the following aspects:

1) Selection bias: sequence generation, baseline characteristics, and allocation concealment.
2) Performance bias: random housing and blinding of caregivers and/or investigators.
3) Detection bias: random outcome assessment and blinding of the outcome assessor.
4) Attribution bias: incomplete outcome data.
5) Reporting bias: selective outcome reporting.
6) Other sources of bias.

The bias grade of each field was classified as high, low, or unclear risk. Any disagreements were discussed and resolved with a third assessor.

### 2.6 Statistical analysis

Following the Cochrane Handbook for Systematic Reviews of Interventions (Higgins et al., 2019), the overall effects of all continuous variables were reported by weighted mean difference (MD) with the 95% confidence interval (CI), and *p* values <0.05 were defined as indicative of statistical significance. The heterogeneity of multiple results was tested by performing the Chi-square (Chi^2^) and I-square (I^2^) tests. For the Chi^2^ test, the level of α = 0.1 was considered to be indicative of statistical significance, and for the I^2^ test, the level was I^2^ = 50%. More specifically, a fixed-effects model was used for I^2^ < 50%; otherwise, a random-effects model was applied. To explore potential sources of heterogeneity and how they affected the pooled effects, subgroup analyses were performed depending on the modeling methods of CHF [drug: doxorubicin (DOX); surgery: transverse aortic constriction (TAC), abdominal aorta constriction (AAC), left anterior descending coronary artery ligation (LAD)] and durations (short: < 21 days; long: ≥ 21 days). For studies with medium or high heterogeneity, sensitivity analyses were organized to investigate the influence of each study by omitting studies one by one when there were adequate studies. Publication bias and small-study effects were assessed by funnel plots and Egger’s test on the condition that ≥10 trials were included for an outcome. The results of Egger’s test were considered to be associated with considerable publication bias or small-study effects when *p* values were <0.05 (Egger et al., 1997). This meta-analysis was performed in RevMan V.5.3 software, whereas subgroup analysis, sensitivity analysis, and the evaluation of publication bias were performed in STATA/SE 15.1 software.

## 3 Result

### 3.1 Study search

**FIGURE 1:**
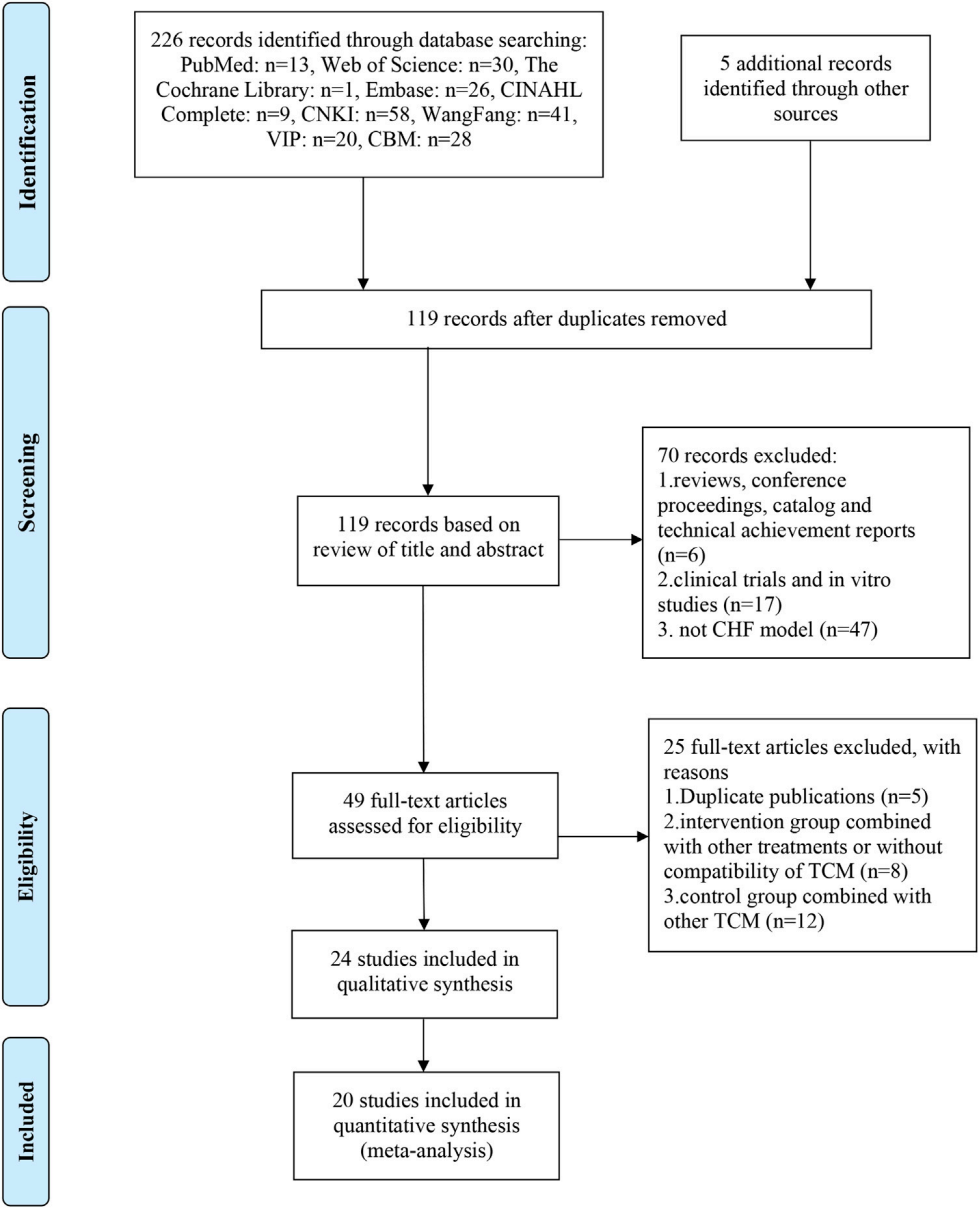
PRISMA flow chart of study search process.

As shown in Figure 1, a total of 226 records were identified according to the established search strategy in ***2.1*** and 5 records were identified through manual searching. After eliminating duplicates, 119 records were left for screening based on titles and abstracts, 70 of which were excluded for listed reasons. Then, 49 full-text articles were assessed for eligibility, and 25 of them were excluded based on selection criteria. Eventually, 24 studies (Yang et al., 2013; Zhai et al., 2013; Chen S. et al., 2014; Liang et al., 2014; Jin et al., 2015; Miao et al., 2015; Xu, 2015; Miao et al., 2016; Wang et al., 2016; Xu et al., 2016; Wan et al., 2017; Fu et al., 2018; Peng, 2018; Sun et al., 2018; Wu, 2018; Jia et al., 2019; Sun et al., 2019; Wen et al., 2019; Yang, 2019; Wen et al., 2020a; Wen et al., 2020b; Yan et al., 2020; Xie et al., 2021; Ni et al., 2022)were included in the qualitative synthesis, and 20 of them were included in the quantitative synthesis (meta-analysis).

### 3.2 Study characteristics

Twenty-three studies involving 686 animals, with 445 in the intervention group and 241 in the control group, were included in the systematic review and meta-analysis, and 1 study did not report the number of participating animals. Twenty-two trials chose rats as the experimental subjects, whereas two chose mice, mostly males. The weights of the rats ranged from 160 to 300 g, but 1 study used mice weighing from 20 to 24 g. For modeling methods, drug inducement and surgery were the major choices, and DOX-inducement was the most frequently used (13 of 24 studies, 54.2%), with LAD and TAC used less frequently (4 of 24 studies, 16.7% each). The use of AAC was mentioned in only three (12.4%) studies, which was the least. Interventions involved in all studies were mainly Fuzi or its bioactive constituents, including AC, HA, higenamine (HG), hei-shun-pian, and total alkaloids (TA) that were compatible with ginseng, red ginseng, shan-zhu-yu (*Cornus officinalis Sieb.* et Zucc.), banxia (*Pinellia ternate* (Thunb.) Breit.), fuling (*Poria cocos*), gancao (*Glycyrrhizae Radix* et Rhizoma) or its bioactive constituents, including glycyrrhetinic acid (GA) and liquiritin, dried ginger (*Zingiberis* Rhizoma) or its bioactive constituents, including [6]-gingerol ([6]-GR) and SND or its components, including total alkaloids and total gingerols (TAG), total alkaloids, flavones, and saponins (TAFS), and total alkaloids, total gingerols, total flavones, and saponins (TAGFS). In contrast, the administrations in the control groups were only of Fuzi or its bioactive constituents. Moreover, the duration of each study was noted and divided into short- or long-duration groups based on the average duration (21 days), with 10 studies in the short group, 13 in the long, and 1 without duration reported. The outcome indexes included in each study and all of the above characteristics of the studies are presented in Supplementary Material, Supplementary Table S1.

### 3.3 Study quality

The results of the quality assessment are presented in Supplementary Material, Supplementary Table S2.

1) Selection bias: random allocation to the control group and intervention group were mentioned in all included studies, of which three studies described the detailed method of random sequence generation, referring to a random number table. The animals’ baseline characteristics (sex, age, and weight) of each trial were similar in both the control and intervention groups, and the timing of CHF induction was also adequate. None of the studies mentioned allocation concealment. As a result, three in the 24 studies were identified as having low risks of bias in sequence generation, and all studies were assessed to have low risks of bias in the baseline characteristics and unclear risks of bias in allocation concealment.
2) Performance bias: none of the studies reported if the animals were randomly housed during the experiment or not or if the caregivers and investigators were blinded, so the assessment of random housing and blinding of this item were identified as unclear risks of bias in all studies.
3) Detection bias: none of the studies described randomly picking an animal for outcome assessment or using a random component in the sequence generation for outcome assessment as well as the blinding of outcome assessors, which indicated unclear risks of bias in all studies in random outcome assessments and blinding of this aspect.
4) Attribution bias: all animals were included in the analysis, and no outcome data were missing from any of the studies, so for incomplete outcome data, low risks of biased data were judged for all studies.
5) Reporting bias: it was clear that all published reports included all expected outcomes and comparative methods, and results sections, suggesting low risks of bias in selective outcome reporting.
6) Other sources of bias: for this aspect, funding was the main factor considered, and 19 studies reported that their studies were free of the inappropriate influence of funders, whereas 5 did not, resulting in 19 studies at low risks of bias in funding and 5 at unclear risks of bias.

### 3.4 Effectiveness

A total of 12 outcome indexes were identified as sufficient for meta-analysis, including BNP, HR, HWI, ALD, LVEDP, LVSP, EF, FS, +dP/dt_max_, −dP/dt_max_, TNF-α, and the activity of Na ^+^ -K ^+^ -ATPase. Meta-analysis was not performed for the other outcomes due to insufficient data.

#### 3.4.1 BNP

**FIGURE 2:**
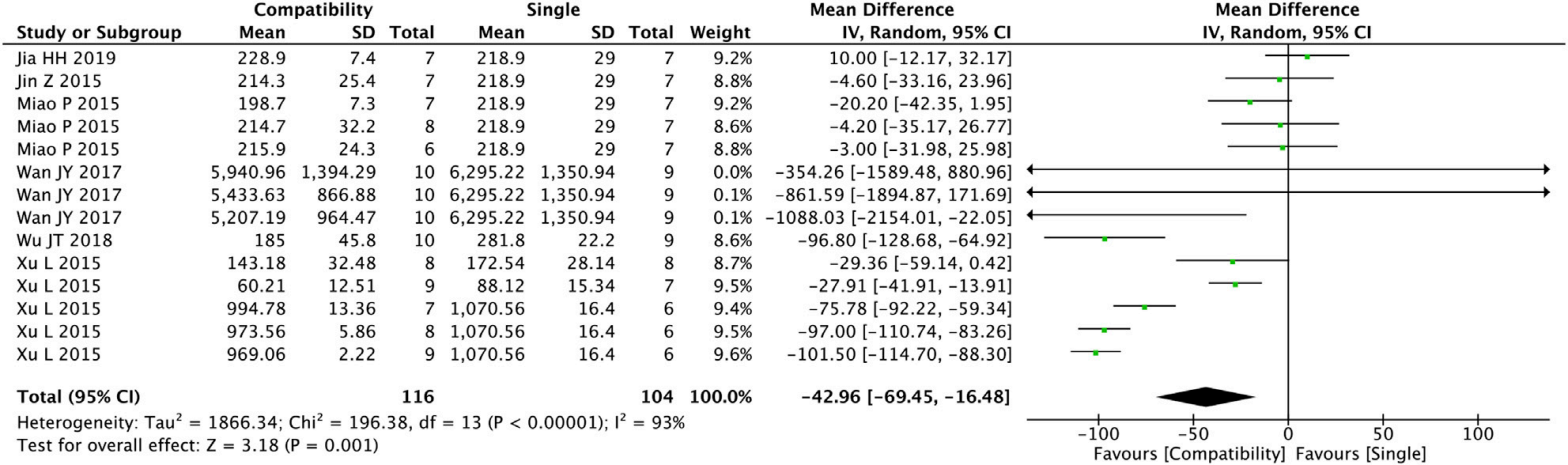
Forest plot of BNP.

As shown in Figure 2, a total of 14 studies were included to assess the general compatibility effect of Fuzi on BNP. Statistical tests revealed a significant difference between the two groups (MD = −42.96, 95%CI [−69.45, −16.48], *p* = 0.001), indicating a significant effect on decreasing the level of BNP in the compatible group compared with the control group, but with a high heterogeneity (Chi^2^ = 196.38, *p* < 0.00001; I^2^ = 93%).

#### 3.4.2 HR

**FIGURE 3:**
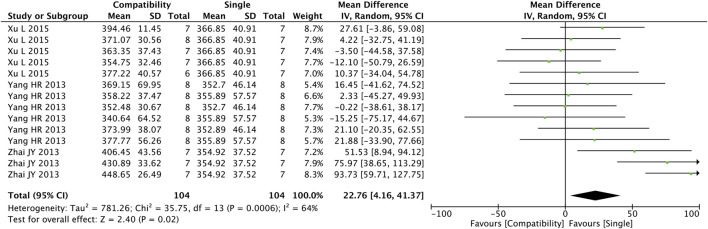
Forest plot of HR.

Figure 3 shows the results obtained from the preliminary analysis of HR. Fourteen studies reported the compatibility effect of Fuzi on HR, and a significant difference was found in the compatibility group when compared with the control group (MD = 22.76, 95%CI [4.16, 41.37], *p* = 0.02), with a medium heterogeneity (Chi^2^ = 35.75, *p* = 0.0006; I^2^ = 64%), suggesting that the compatibility group increased the HR of the CHF patients.

#### 3.4.3 HWI

**FIGURE 4:**
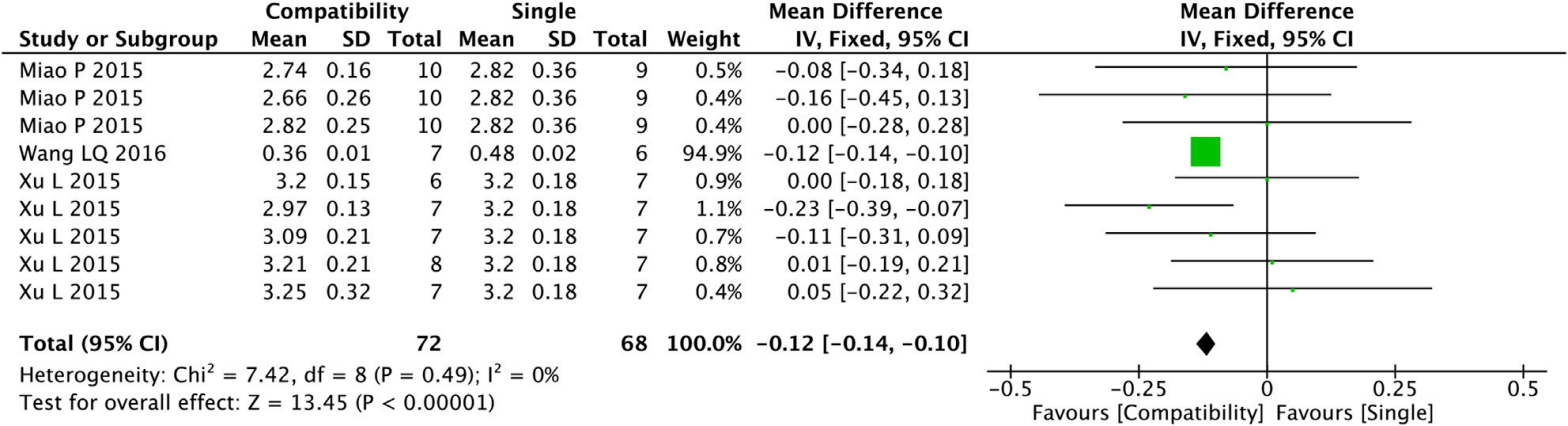
Forest plot of HWI.

Figure 4 shows that nine studies were included in the analysis of the compatibility effect of Fuzi with other treatments on HWI altogether. A more valid correlation was found between compatibility and HWI drop compared with the control group (MD = −0.12, 95%CI [−0.14, −0.10], *p* < 0.00001), with a low heterogeneity (Chi^2^ = 7.42, *p* = 0.49; I^2^ = 0%).

#### 3.4.4 ALD

**FIGURE 5:**
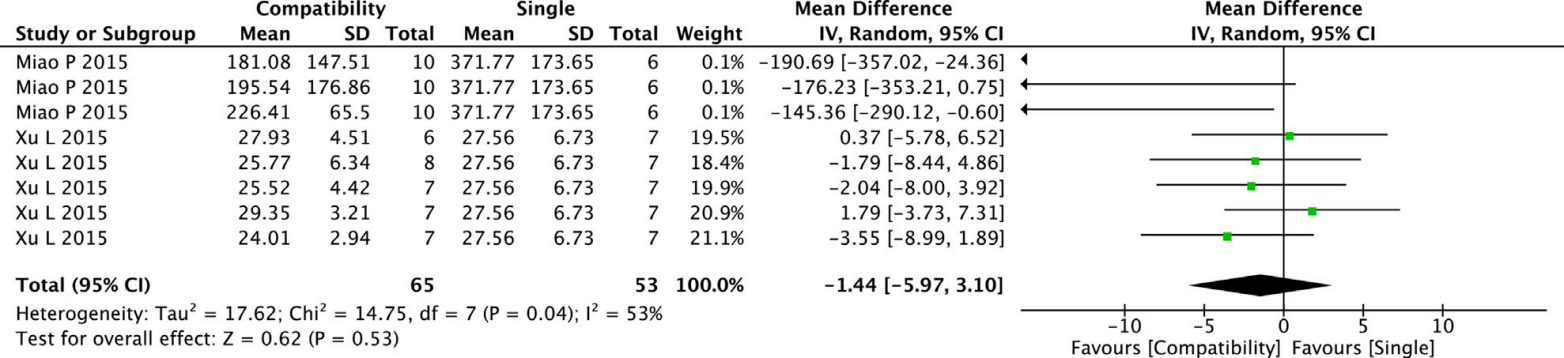
Forest plot of ALD.

The pooled results of the compatibility effect of Fuzi with other treatments on ALD are shown in Figure 5. Eight trials were included in this comparison. The results showed a slight decrease in ALD in the compatibility group but the difference between two groups was not significant (MD = −1.44, 95%CI [−5.97, 3.10], *p* = 0.53), with medium heterogeneity (Chi^2^ = 14.75, *p* = 0.04; I^2^ = 53%).

#### 3.4.5 LVEDP and LVSP

**FIGURE 6:**
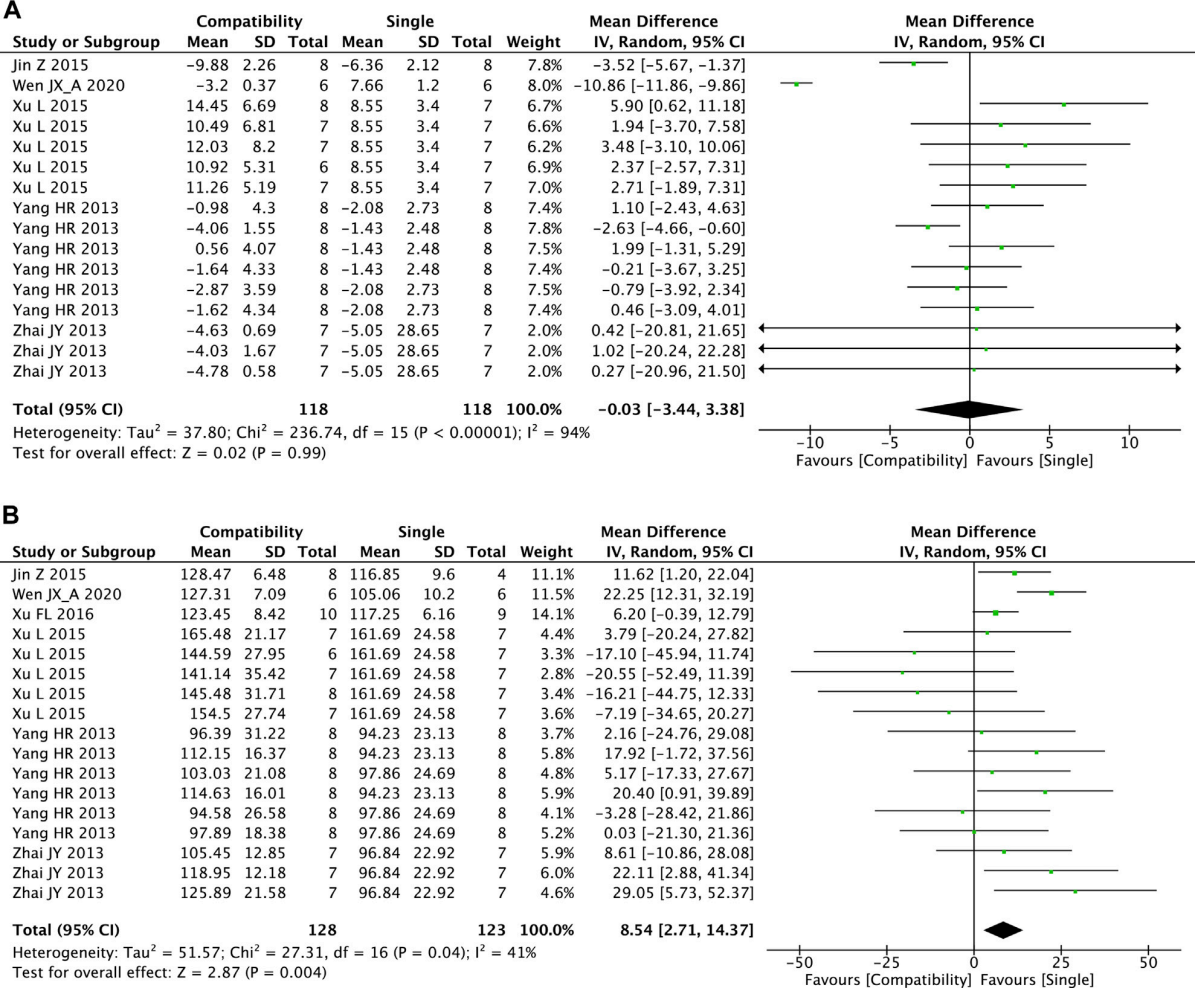
Forest plot of LVEDP and LVSP.

Figures 6A,B shows that there were 16 and 17 studies included in the meta-analysis of the effect of Fuzi compatibility with other treatments on LVEDP and LVSP, respectively. Although the pooled result showed slight decreases in LVEDP, the difference between two groups was not significant (MD = −0.03, 95%CI [−3.44, 3.38], *p* = 0.99) and with high heterogeneity (Chi^2^ = 236.74, *p* < 0.00001; I^2^ = 94%). For LVSP, the overall effect test showed the significant superiority of the compatibility group in increasing LVSP versus the control group (MD = 8.54, 95%CI [2.71, 14.37], *p* = 0.004) with medium heterogeneity (Chi^2^ = 27.31, *p* = 0.04; I^2^ = 41%).

#### 3.4.6 EF and FS

**FIGURE 7:**
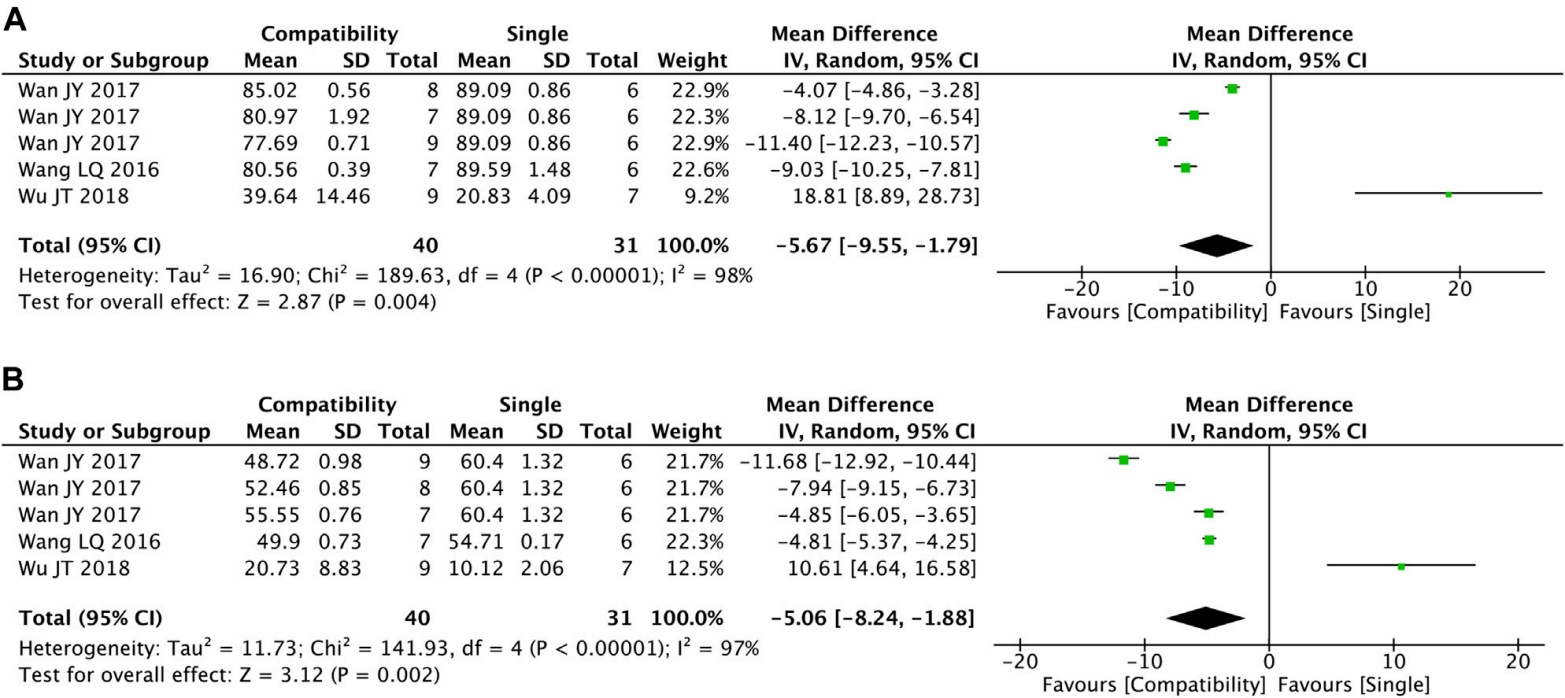
Forest plot of EF and FS.

Figures 7A,B shows the summary statistics of EF and FS from five trials for each. It was apparent that the compatibility group was significantly superior in decreasing the two indexes versus the control group (EF: MD = −5.67, 95%CI [−9.55, −1.79], *p* = 0.004; FS: MD = −5.06, 95%CI [−8.24, −1.88], *p* = 0.002), with high heterogeneity for both (EF: Chi^2^ = 189.63, *p* < 0.00001; I^2^ = 98%; FS: Chi^2^ = 141.93, *p* < 0.00001; I^2^ = 97%).

#### 3.4.7 + dP/dt_max_ and −dP/dt_max_

**FIGURE 8:**
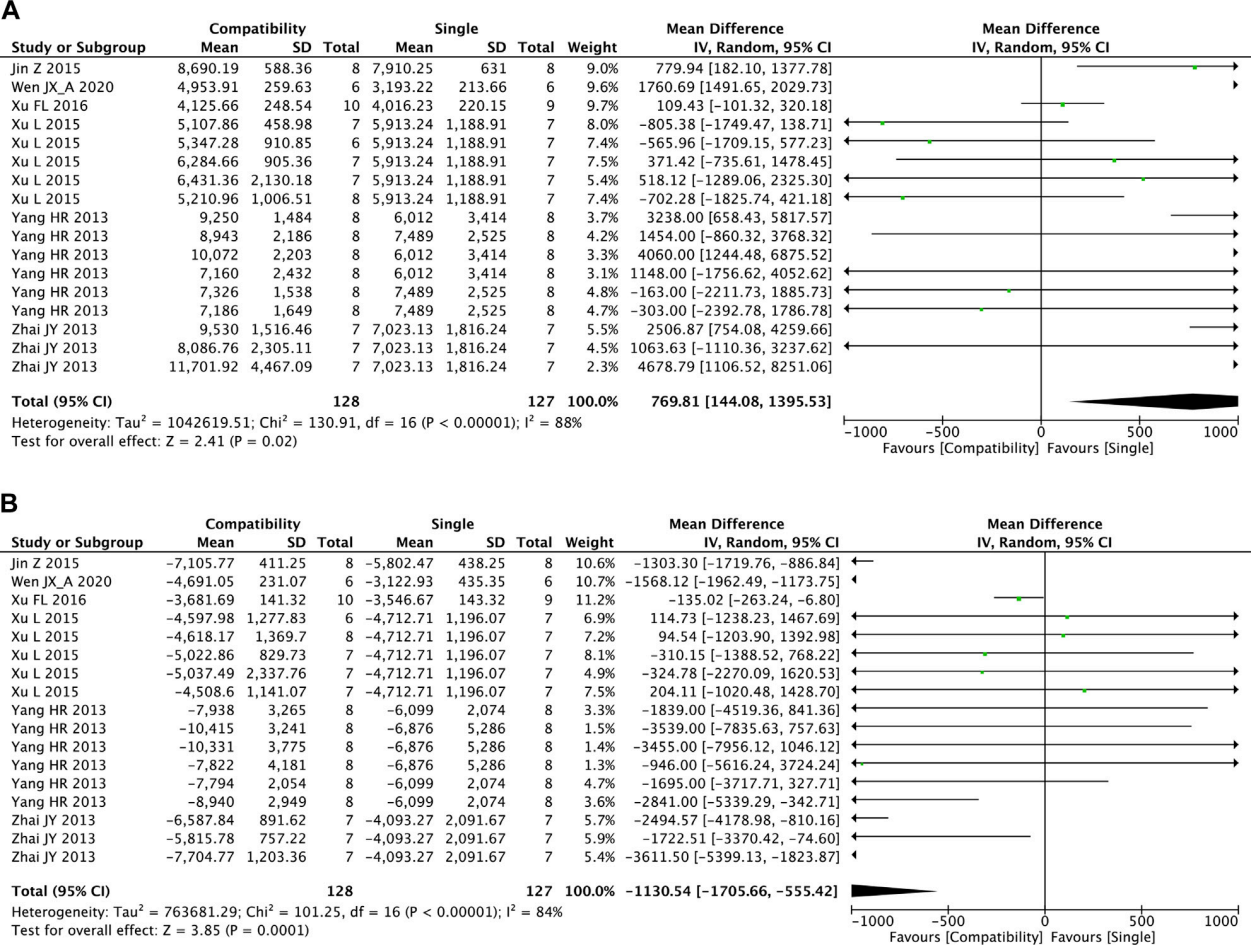
Forest plot of +dP/dt_max_ and −dP/dt_max_.

The overall effects of Fuzi compatibility on + dP/dt_max_ and −dP/dt_max_ are shown in Figures 8A,B, with 17 studies included for each. The pooled effect of Fuzi increased + dP/dt_max_ but decreased −dP/dt_max_ relative to the control group. The compatibility group was able to increase both the maximum increase rate and decrease rate of left ventricular pressure significantly (+dP/dt_max_: MD = 769.81, 95%CI [144.08, 1395.53], *p* = 0.02; −dP/dt_max_: MD = −1130.54, 95%CI [−1705.66, −555.42], *p* = 0.0001). Both + dP/dt_max_ and −dP/dt_max_ showed high heterogeneity (+dP/dt_max_: Chi^2^ = 130.91, *p* < 0.00001; I^2^ = 88%; −dP/dt_max_: Chi^2^ = 101.25, *p* < 0.00001; I^2^ = 84%).

#### 3.4.8 TNF-α

**FIGURE 9:**
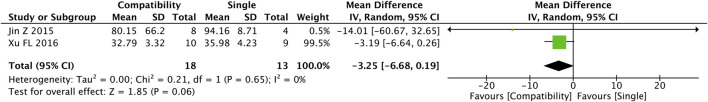
Forest plot of TNF-*α*

Figure 9 shows the results of the pooled Fuzi compatibility effect on TNF-α. Only two studies reported on this index with analyzable data, and the compatibility group showed a decrease in the level of TNF-α versus that in the control group, but the difference was not significant (MD = −3.25, 95%CI [−6.68, 0.19], *p* = 0.06) and the heterogeneity was low (Chi^2^ = 0.21, *p* = 0.65; I^2^ = 0%).

#### 3.4.9 Na + -K+-ATPase

**FIGURE 10:**
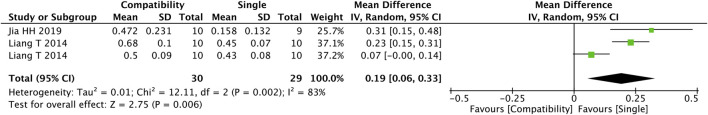
Forest plot of Na^+^-K^+^-ATPase.

Figure 10 shows that three studies were included in the analysis of the Fuzi compatibility effect on the activity of Na ^+^ -K ^+^ -ATPase. The compatibility group improved the activity of Na ^+^ -K ^+^ -ATPase significantly versus the control group (MD = 0.19, 95%CI [0.06, 0.33], *p* = 0.006), with high heterogeneity (Chi^2^ = 12.11, *p* = 0.002; I^2^ = 83%).

### 3.5 Subgroup analysis

Subgroup analyses were performed depending on two factors: the modeling methods of CHF and duration. For CHF modeling methods, four were mentioned, including DOX, TAC, AAC, and LAD, in keeping with the four kinds of subgroups. Duration, the average of which (21 days) was used to classify the subgroups into short- (<21 days) and long- (≥21 days) duration subgroups. The outcome indexes of BNP, HR, HWI, ALD, LVEDP, LVSP, +dP/dt_max_, and −dP/dt_max_ were selected for subgroup analyses whereas the other outcomes were excluded due to insufficient studies. The results of subgroup analysis can be found in Supplementary Material, Supplementary Tables 3–10.

#### 3.5.1 BNP

The subgroup analysis results for BNP are presented in Supplementary Table S3. Among the modeling methods, there were significant differences in the efficacy of Fuzi compatibility in the AAC (*p* = 0.000), TAC (*p* = 0.013), and LAD (*p* = 0.000) subgroups, but not in the DOX subgroup (*p* = 0.523), indicating that the outcome in DOX group was not credible though it presented to be positive. The efficacy of Fuzi compatibility was significant in the two duration subgroups (short: *p* = 0.013; long: *p* = 0.002). However, the two factors were not the source of its high heterogeneity.

#### 3.5.2 HR

As shown in Supplementary Table S4, the subgroup analyses for HR found that there was a significant difference in the efficacy of Fuzi compatibility in the DOX group (*p* = 0.015) but not in the AAC group (*p* = 0.388) versus the control group, suggesting that the results of the experiment on HR in the AAC group were invalid. In addition, significant compatible efficacy was shown in the short-duration subgroup (*p* = 0.015) but not in the long-duration subgroup (*p* = 0.388), which was proof of the suspect outcome of the experiment on HR in the long-duration group. Neither of the two factors was the source of heterogeneity.

#### 3.5.3 HWI

The results of subgroup analyses on HWI are presented in Supplementary Table S5. For modeling methods, significant efficacy of compatibility was only shown in the TAC group (*p* = 0.000) and not in the DOX (*p* = 0.323) and AAC (*p* = 0.185) groups, indicating that the results for HWI were not valid in the DOX and AAC groups. Moreover, duration did not have a significant effect on the pooled compatibility effect (short: *p* = 0.000; long: *p* = 0.045). A heterogeneity test was not performed for this outcome index due to its low heterogeneity.

#### 3.5.4 ALD

For ALD, subgroup analysis was performed only for the modeling methods with sufficient studies on the duration (Supplementary Table S6). There was a significant difference in the Fuzi compatibility efficacy between the DOX group (*p* = 0.000) but not between the AAC group (*p* = 0.440) and the control group, which indicated that the modeling method was a key factor affecting the overall results. Further, the heterogeneity results of ALD suggested that the modeling method was the source of its heterogeneity (**^**).

#### 3.5.5 LVEDP and LVSP

For LVEDP, no significant associations between the efficacy of compatibility and LVEDP in the modeling method subgroups (DOX: *p* = 0.332; AAC: *p* = 0.543) and durations (short: *p* = 0.312; long: *p* = 0.998) were found (Supplementary Table S7). The results of subgroup analyses on LVSP are shown in Supplementary Table S8. Depending on the modeling method, the efficacy of compatibility was significant in the DOX group (*p* = 0.000) but not in the AAC group (*p* = 0.599). The short-duration subgroup showed significant efficacy of compatibility (*p* = 0.001), which was not observed in the long-duration subgroup (*p* = 0.400). Interestingly, the pooled effects of the DOX and AAC subgroups in the subgroup analyses of both LVEDP and LVSP were opposite from each other, which was also observed for the short- and long-duration subgroups. Neither of the grouping bases led to LVEDP’s high heterogeneity, whereas the modeling method was shown to be the source of LVSP heterogeneity (**^**).

#### 3.5.6 +dP/dt_max_

Supplementary Table S9 shows the results of subgroup analyses on the efficacy of compatibility on +dP/dt_max_. A significant was found for the DOX subgroup (*p* = 0.001) but not in the AAC subgroup (*p* = 0.868). Significant efficacy of compatibility was observed for the short-duration subgroup (*p* = 0.002) but not in the long-duration subgroup (*p* = 0.559). Although the efficacy results were opposite in the DOX and AAC subgroups, the two factors were not the source of high heterogeneity for +dP/dt_max_.

#### 3.5.7 −dP/dt_max_

Supplementary Table S10 shows the results of subgroup analyses on the efficacy of compatibility on −dP/dt_max_. There was a significant effect on efficacy in the DOX subgroup (*p* = 0.000) but not in the AAC subgroup (*p* = 0.289). Significant efficacy of compatibility was observed for the short-duration subgroup (*p* = 0.000), but not in the long-duration subgroup (*p* = 0.121). The test of heterogeneity showed that the two factors were not the source of high heterogeneity for *−*dP/dt_max_.

### 3.6 Sensitivity analysis

Sensitivity analyses were performed on BNP, HR, ALD, EF, FS, LVEDP, LVSP, +dP/dt_max_, −dP/dt_max_, and the activity of Na + -K + -ATPase by omitting studies one by one to investigate how each study influenced the pooled effects. No sensitivity analysis was performed on the other outcome indexes due to insufficient studies. After successively excluding each of the studies, the combined results of BNP, HR, ALD, LVEDP, LVSP, +dP/dt_max_, and −dP/dt_max_ did not change substantially, indicating that although their heterogeneity was high, the results were stable and credible. However, the sensitivity analyses on EF, FS, and Na + -K + -ATPase showed changes when certain studies were omitted, and the pooled effects varied from those for the primary analysis and were no longer statistically significant, which indicated that some of the included studies influenced the overall evaluation. Supplementary and further information about the sensitivity analyses are provided in Supplementary Material, Supplementary Appendix S2, Supplementary Figures 11–20.

### 3.7 Publication bias and small-study effect

Publication bias and the small-study effect were evaluated through funnel plots and Egger’s test on BNP, HR, LVEDP, LVSP, +dP/dt_max_, and −dP/dt_max_ but not on the other outcome indexes because they had <10 included studies. Supplementary Figures F1–F6 show the funnel plots, and various degrees of asymmetry can be seen in all funnel plots. The results of Egger’s test are shown in Supplementary Figures E1–E6. The publication bias and small-study effect in LVEDP (bias: 4.71, 95%CI [2.41, 7.01], *p* = 0.001) and −dP/dt_max_ (bias: −1.58, 95%CI [−2.94, −0.22], *p* = 0.026) were significant. All supplementary materials for the publication bias and small-study effect assessment are detailed in Supplementary Material, Supplementary Appendix S3.

## 4 Disscusion

### 4.1 Mechanisms of synergistic compatibility

#### 4.1.1 Improvements in cardiac function

##### 4.1.1.1 Hemodynamic process

Hemodynamics analyses are considered the most accurate among a series of cardiac function tests and an essential assessment of heart condition in rats (Khorrami et al., 2014). HR represents the number of times the heart ventricles contract per unit of time. For CHF rats, HR will rise due to conditioned reflexes to make up for heart-induced insufficient blood supply (Huff et al., 1999). LVSP and LVEDP mainly relate to myocardial contraction and diastole. The ±dP/dt_max_ parameter evaluates the rate of left ventricular pressure and degree of myocardial diastole, which is susceptible to cardiac load, and also represents the ability of myocardial contraction and diastole. Hence, the above five outcome indexes are classic parameters for hemodynamic analyses, directly reflecting an animal’s blood flow state. For animals in a CHF model, their LVSP and +dP/dt_max_ will drop dramatically, whereas LVEDP and −dP/dt_max_ will rise significantly, indicating a prominent decrease in myocardial contractility, left ventricular overfilling, and impaired diastolic function (Thibault et al., 2013). The present meta-analysis results showed that the synergistic effect of Fuzi compatibility with other reviewed study treatments increased HR, LVSP, and +dP/dt_max_ as well as decreased −dP/dt_max_ relative to the effect of Fuzi used alone. Although there was no significant difference in the pooled effect of LVEDP, some individual studies showed decreases. All things considered, the mechanism of Fuzi synergistic compatibility on hemodynamics is likely to be enhancement of myocardial contraction and diastolic ability, increased coronary blood flow, reduction of myocardial oxygen consumption, improvement of myocardial cellular energy metabolism, and alleviation of heart load, all of which would inhibit further deterioration of HF.

##### 4.1.1.2 Heart pumping function and cardiac contractility

Echocardiography is one of the important techniques commonly used to detect heart function and can be used clinically to evaluate the severity of HF. Common indicators of cardiac structural and functional changes are EF and FS (Wan et al., 2017). FS refers to the left ventricular (LV) short-axis shortening rate, and its normal reference range is 25%–45% (average: 30%). The EF is more commonly used clinically. Left ventricular EF (LVEF) refers to stroke volume as a percentage of ventricular end-diastolic volume. When the ventricles contract, all of the blood in the ventricles cannot be injected into the arteries. In a normal adult resting state, the diastolic volume of the left ventricle is about 125 ml, and the stroke volume is 60–80 ml. Generally, 55%–75% is considered the normal range. When the human body is at rest, the EF is about 55%–65% and is related to myocardium contractility; specifically, stroke volume and EF increase with stronger myocardial contractility. Under normal circumstances, LVEF is ≥50%, and <50% is regarded as insufficiency of LV contractility. Clinically, CHF patients are classified as HF with reduced EF (HFrEF: EF < 40%), HF with mid-range EF (HFmrEF: EF 40%–49%), and HF with preserved EF (HFpEF: EF ≥ 50%) (Iwano and Little, 2013; Tschöpe et al., 2019). In this paper, the levels of both EF and FS decreased and returned to the relatively normal range in the Fuzi compatibility group but not in the Fuzi alone control group, suggesting that there were improvements in the cardiac pumping function and enhancement of cardiac contractility with Fuzi compatibility. Reverse cardiac remodeling to some extent was also implied.

#### 4.1.2 Improvements in ventricular remodeling and cardiac damage

HF mainly damages the myocardium, including ventricular overload during diastole and systole, which changes the number of myocardial cells (myocardial infarction, diffuse myocarditis) that causes atrial and ventricular hypertrophy. Pathological cardiac hypertrophy is associated with cardiac insufficiency and is usually induced by many factors, such as prolonged and abnormal hemodynamic stress, activation of pro-inflammatory cytokines, and cellular dysfunction, and all these complicated responses cause cardiac remodeling changes and potentially CHF (Ma et al., 2016; Shimizu and Minamino, 2016). According to the results of hematoxylin and eosin staining on the cardiac tissue of CHF rats, pathological changes were found that compared with the normal group, the HWI of the CHF group increased, with obvious myocardial hypertrophy, myocardial tissue infarction, myocardial tissue cell death area, myocardial layer thickening, and myocardial fibrous space widening (Xie et al., 2021). HWI refers to the relative value of heart weight and body weight, which reflects the degree of cardiac remodeling and hypertrophy. As an evaluation of heart function, its increase is often accompanied by obvious myocardial hypertrophy or myocardial tissue infarction (Xu, 2015). LDH is an important enzyme in the glycolysis pathway, widely presented in various tissues of the human body. The content of LDH in red blood cells is about 100 times that of normal serum and is abundant in heart and lung tissues. LDH reversibly catalyzes the oxidation reaction of lactic acid into pyruvic acid and catalyzes the oxidation of lactic acid to propionic acid while simultaneously transferring hydrogen to the coenzyme to become NADH. Due to LDH’s wide distribution, its specificity for disease diagnosis is poor. However, because of the large difference in enzyme content between tissue and plasma, it has high sensitivity. When HF occurs, the activity of LDH is hundreds of times higher in myocardial cells than in serum, so even the slightest damage to the myocardium causes a significant increase in serum LDH activity. CK is an important enzyme in human energy metabolism and is mainly distributed in skeletal muscle and myocardium, followed by brain tissue. The activity of CK increases within 3–8 h after myocardial injury, peaks at 12–36 h, and returns to the level in healthy people in 3–5 days (Li et al., 2001). Continuous increase in CK activity indicates more severe myocardial damage. LDH and CK are used as myocardial injury markers whose activity changes can be dynamically monitored to determine the degree of HF and observe treatment efficacy and predict prognosis. Chen S. et al. (2014) found that the levels of LDH and CK in mice were elevated after DOX inducement. However, when administered with SND and preparations containing TA, both the levels of LDH and CK were improved to normal ranges, which was not observed with TA alone, suggesting the compatibility synergistic effect. Wen et al. (Wen et al., 2019) measured the activities of myocardial damage markers, including LDH and CK, to determine whether heart damage had occurred as reflected in serum biomarkers, and the results showed that the marker levels increased significantly in the DOX-inducement model group but were reduced in the compatibility group, which was the combination of Fuzi and dried ginger. This meta-analysis showed a significant decrease in the level of HWI in the compatibility group relative to that in the control group as reflected by the LDH and CK levels, suggesting that the addition of Fuzi improved the efficacy via synergistic mechanisms involved in reversing ventricular remodeling, resisting cardiac hypertrophy, and alleviating cardiac damage.

#### 4.1.3 Increase in the activity of Na ^+^ -K ^+^ -ATPase and Ca^2+^ overload suppression

The heart is one of the main energy-consuming organs in the human body, and energy metabolism is the material basis of myocardial cell activity, of which the tricarboxylic acid cycle is the main process. Under normal circumstances, mitochondria are the primary sites for myocardial energy production and storage. ATP is an important guarantee for maintaining cardiac contractile function and energy metabolism, 60%–90% of which comes from the oxidation of fatty acids, and the rest comes from carbohydrates (sugar and lactic acid) and a small amount of ketone oxidation (Stanley et al., 1997). The continuous and rhythmic contraction and relaxation of normal cardiomyocytes require a steady stream of ATP to provide energy for their activities. Disorders in the metabolism of fatty acids, glucose, lactic acid, and amino acids in myocardial cells during HF can cause changes in cardiac energy metabolism, leading to myocardial contraction-coupling disorder and progression of the ventricular remodeling (van Bilsen et al., 2004). Current research suggests (Chen Y. et al., 2014) that when HF occurs, the balance between oxygen supply and oxygen consumption is broken, and mitochondrial function is changed from the use of fatty acids to greater use of glucose, phosphorylation potential, as well as ATP production and utilization, are reduced, and the AMP and ADP contents increase. Consequently, the combination of ventricular remodeling accompanied by energy metabolic disorders is considered to be the main pathophysiological mechanism of CHF, which has been confirmed by the detection of high-energy phosphoric acid compounds in the CHF myocardium (Neubauer, 2007). Na ^+^ -K ^+^ -ATPase, also known as the sodium pump or sodium–potassium pump, is mainly used in energy supply, elimination of intracellular waste, and maintaining cell-pressure balance. Currently, studies have found that when the activity of the sodium-potassium pump on myocardial cells continues or severely decreases, it will cause severe dysfunction of the sodium pump, leading to abnormal myocardial energy metabolism, heart damage, or cardiac insufficiency (Huang, 2013). Na ^+^ -K ^+^ -ATPase is widely distributed on the cell membrane. In the pathophysiological process of CHF, the change in Ca^2+^ regulation is the central link between various mechanisms. The protein level and activation degree of Na ^+^ -K ^+^ -ATPase in the myocardial cell membrane are the key factors of intracellular Ca^2+^ homeostasis. Studies have shown that when CHF occurs, Na ^+^ -K ^+^ -ATPase activity decreases, intracellular Na ^+^ increases, and Na ^+^ -K^+^ exchange increases, leading to mitochondrial dysfunction and reduced ATP production. Plasma and sarcoplasmic membranes that rely on energy Ca^2+^-ATPase cannot pump out or take in the excess Ca^2+^ into the sarcoplasmic reticulum due to insufficient energy, causing calcium overload in myocardial cells (Zhu, 2012). Miao et al. (Miao et al., 2016) found that in the state of CHF, the content of ATP in rat myocardial cells was significantly reduced, whereas that of AMP and ADP was increased. After intervention with SND, a significant increase in the content of ATP was found in the myocardium of rats with CHF, indicating that improving myocardial cell energy metabolism may be one of the important curative mechanisms of SND and its different compatible prescriptions for myocardial protection. In addition, our meta-analysis results on the activity of Na ^+^ -K ^+^ -ATPase showed that the compatibility of Fuzi improved its activity relative to that of Fuzi alone, providing evidence for Fuzi’s synergistic compatibility mechanisms on energy metabolism, which include increasing the activity of Na ^+^ -K ^+^ -ATPase on myocardial cells, regulating myocardial energy metabolic disorders, and ultimately enhancing myocardial cell activity and heart pump function.

#### 4.1.4 Regulation of the renin–angiotensin–aldosterone system

The renin-angiotensin-aldosterone system (RAAS) is a vital human body system because it maintains plasma sodium concentration, arterial blood pressure, and extracellular volume. Kidney-secreted renin enzyme acts on its substrate to form Ang II, a versatile effector peptide hormone (Patel et al., 2017), which is the main medium for RAAS to achieve its biological effects. ALD is a hormone that regulates blood volume in the human body, and the level is positively correlated with the severity of HF. Many experiments have confirmed that the RAAS and renin-angiotensin system (RAS) are activated during HF, in which ALD is particularly important, directly reflecting the effects of drugs in the treatment of HF. Elevating ALD in a short period in HF can increase cardiac output and thus have a compensatory effect, but if ALD continues to increase long term, it can cause water and sodium retention, increase blood volume of the circulatory system, and promote ventricular preload and myocardial cell fibrosis (McMahon, 2001), causing malignant arrhythmia or sudden death. Studies have confirmed that the reduction in ALD can reduce ventricular load and reduce the responsiveness of blood vessels to sympathetic nerves, thereby directly dilating blood vessels and improving heart function. Normally, Ang II can maintain vascular tension, excite sympathetic nerves, and regulate ALD secretion. However, under pathological conditions, Ang II becomes the main factor leading to ventricular remodeling and stimulates the increase in ALD secretion. Simultaneously, the increased release of ALD causes myocardial cell apoptosis, myocardial ischemia, and arrhythmia promoting ventricular remodeling and inducing and aggravating HF (Xiong and Wang, 2013), forming a vicious cycle. ET-1, a strong vasoconstrictor in the body, is secreted by vascular endothelial cells and cardiomyocytes. ET-1 can promote the synthesis and release of Ang II in cardiomyocytes and vascular smooth muscle cells as well as promote the proliferation of cardiac fibroblasts and synthesis of collagen. Studies have found that CHF can be accompanied by increased ET-1 levels (Xie and Peng, 2013). BNP is a peptide hormone of the natriuretic peptide class. Studies have found that the content of BNP is much higher in human heart tissue than in brain tissue. The BNP content is highest in the right and left atria of the human body. Ventricular volume load and ventricular wall tension affect the synthesis and secretion of BNP, so the concentration of BNP in serum can be used to reflect ventricular function (Xu, 2015). When HF occurs, the increase in LV filling pressure and ventricular wall tension are the key reasons for the increase in BNP secretion (Miao et al., 2015). Studies (Wang et al., 2012) have confirmed that with the gradual aggravation of HF, the level of BNP also increases, and when improvements in heart function are made, the level of BNP will significantly decrease, suggesting that BNP can be used as a marker to observe the severity of HF and assess the risk stratification and prognosis of HF. In addition, BNP, as a cardiac neuroendocrine hormone secreted by ventricular cardiomyocytes, is capable of dilating blood vessels, causing diuresis, and inhibiting RAAS and sympathetic nervous system activity (Zhang et al., 2012). Due to the diuretic and vasodilatory effects of BNP, it can resist the effect of RAAS in narrowing blood vessels and effectively prevent myocardial hypertrophy and fibrosis. Therefore, when HF occurs, the BNP plasma concentration is significantly increased; thus, by checking the blood BNP level, the activation condition of the RAAS and the severity of HF can be monitored. In this systematic review, a meta-analysis was conducted on the level of BNP and ALD in CHF rats, and the results showed significant decreases in BNP levels in the compatibility groups versus those in the single utilization groups, whereas for ALD, although there were no significant differences in the overall effect of ALD, decreases could be seen in individual studies. Moreover, it can be concluded from qualitative analyses that the Fuzi compatibility can reduce the levels of ET-1 and AngⅡ in plasma more effectively than using Fuzi alone and achieve a regulatory effect on the RAAS (Fu et al., 2018), proving that the synergistic compatibility mechanism of Fuzi is related to the regulation of RAAS and inhibition of the excessive activation of neuroendocrine factors, thereby enhancing the effect of anti-CHF.

#### 4.1.5 Inhibition of pro-inflammatory cytokines and alleviation on inflammation

Interleukin (IL)-10 is a cytokine that mediates the interaction between leukocytes, and its main role is to inhibit the synthesis and release of inflammatory factors while simultaneously directly inhibiting the activation of inflammatory cells (Iyer and Cheng, 2012), thereby exerting a protective effect on the myocardium (Zhao et al., 2013). IL-18 is mainly produced by activated mononuclear macrophages, which is both a pre-and pro-inflammatory cytokine closely related to various cardiorenal and vascular complications (Bouquegneau et al., 2015), the levels of which are closely related to angina pectoris, myocardial infarction, and fatal coronary events (Schöttker et al., 2013). TNF-α is a multi-dominant cytokine produced by monocytes and macrophages that has a variety of physiological functions, such as regulating the immune response and promoting cell growth and differentiation. The biological activity of TNF-α is transmitted through specific receptors on the cell surface. As an important inflammatory mediator, TNF-α is secreted in large quantities induced by inflammation. Highly expressed TNF-α promotes the differentiation of B cells and improves the activity of B cells, which then promote the synthesis of excess IgE and induce a series of inflammatory reactions. Studies (Wang and Liu, 2014; Ghigo et al., 2014) have shown that increased levels of TNF-α are closely related to myocardial remodeling and decreased ventricular pumping function. CHF is often accompanied by severe inflammation, and myocardial cells can induce a variety of pro-inflammatory factors under chronic stress, including TNF-α, IL-10, and IL-18. Pro-inflammatory factors can activate macrophages through a cascade effect, producing more pro-inflammatory factors and inducing a stronger inflammatory response. Excessive pro-inflammatory factors can both directly inhibit heart function and accelerate the development of HF by inducing myocardial fibrosis and remodeling (Pinchuk et al., 2014). The meta-analysis results for TNF-α provided evidence of the superior therapeutic effects of Fuzi compatibility for decreasing the level of TNF-α in CHF animals relative to the level in single utilization, suggesting that the synergistic compatibility mechanisms of Fuzi involve inhibition of pro-inflammatory cytokines and alleviation of inflammation.

#### 4.1.6 Effect on drug metabolism process *in vivo*

AC and HA are the main active ingredients of Fuzi, and their pharmacokinetics in the body reflect the therapeutic effect of aconite on HF to a certain extent. Through pharmacokinetic experiments, Xie et al. (Xie et al., 2021) found that the absorption of aconite in a CHF rat model was less than normal, elimination was quick, and the retention time in the body was short. After continuous administration of red ginseng, compared with the normal group, the model group showed that AC and HA were mostly eliminated within 24 h, whereas the group administered red ginseng showed almost complete elimination within 48 h, indicating that the absorption of AC in the HF rats increased after compatibility. As a result, the elimination rate was reduced, the working time in the body was prolonged, and the curative effect was enhanced. In addition, preliminary laboratory research (Yang, 2019) found that in normal animals, the compatibility of drug-metabolizing enzymes could produce drug-drug interactions. Among them, the main metabolic CYP450 enzyme subtype of AC and HA was CYP3A4, whose corresponding type in rats was CYP3A2. Further, continuous administration of red ginseng inhibited the activity of CYP3A2 in rats. Further mechanistic studies have found that red ginseng can affect the metabolism of DDAs *in vivo* by regulating the PXR-CYP3A4 pathway. In conclusion, the potential synergistic mechanisms of Fuzi compatibility led to increased absorption of bioactive constituents, a reduced elimination rate so that the *in vivo* duration is prolonged, and the activity of drug-metabolizing enzymes, which affect metabolic behaviours, are inhibited, thus therapeutic effects enhanced.

#### 4.1.7 Inhibition of cardiomyocyte apoptosis

The basic pathogenesis of CHF is ventricular remodeling, which is manifested as heart enlargement and/or myocardial hypertrophy (Cohn et al., 2000). At present, the factors affecting ventricular remodeling mainly include BNP and other neuroendocrine factors as well as the participation in cell apoptosis (Hirota et al., 1999). Among them, cardiomyocyte apoptosis is an important process in the progression of CHF from the compensatory phase to the decompensated phase. Cardiomyocyte apoptosis can cause the hypertrophy of other cardiomyocytes and eventually lead to HF. Apoptosis is the process of autonomous cell death controlled by genes and is an important mechanism for maintaining the normal development and tissue state of the body. Its hyperfunction is closely related to the occurrence of some diseases. Wan et al. (2017) found that the compatibility of GA and liquiritin with HA significantly reduced the expression of B-cell lymphoma-2 (Bcl-2) associated X protein (Bax), caspase-3, fatty acid synthetase (Fas), and Fas-L proteins, and increased expression of Bcl-2 protein. The combination of HA and liquiritin with HA improved more significantly and has a prominent effect on cardiomyocyte apoptosis. Among these effects, the changes in Bcl-2 and Bax were equivalent to the efficacy of the positive control drug digoxin, but the difference was not significant. Simultaneously, GA combined with HA and liquiritin combined with HA significantly reduced the expression of Bax, caspase-3, Fas, and Fas-L protein and increased the expression of Bcl-2 protein relative to those in the HA-single group, which showed an inhibitory effect on cardiomyocyte apoptosis, providing evidence for the potential synergistic mechanisms of Fuzi compatibility providing enhanced inhibition of cardiomyocyte apoptosis, thereby preventing the progression of ventricular remodeling and exerting curative effects in CHF patients.

### 4.2 Implication for further studies

Systematic evaluation of animal experiments is an inevitable trend in the development of evidence-based medicine in human research and animal experiments as well as is a useful tool to improve the quality of animal experiments. Additionally, systematic evaluation is a potential solution to the common problem that current animal experimental results often fail to transform into clinical research. Systematic evaluation of animal experiments can provide a comprehensive and objective understanding of the research status of existing animal experiments and a more reliable basis for screening interventions that can enter clinical trials. Therefore, it is necessary to conduct a rigorous systematic review of animal experiments before clinical trials, which helps avoid waste of both animal and human research resources and prevents putting patients at unknown treatment risks.

The establishment of a CHF model enables further study of the progression of HF and testing of new treatments against it (Sun and Ma, 2016). Although many reliable HF models, such as the use of surgery (e.g., TAC, LAD, AAC) or drug inducements (e.g., DOX) have emerged in recent years with the rapid development of experimental zoology and have made great contributions to clinical trials, drawbacks remain. CHF is a multifactorial systemic disease. After cardiac damage, the structure, neurohumoral, cellular, and molecular mechanisms are activated and serve as a network for maintaining physiological functions. All of these coordinated and complex processes can cause excessive capacity overload, increased sympathetic nerve activities, circulatory redistribution, and lead to different and parallel clinical symptoms (Tanai and Frantz, 2011), resulting in its multifactorial etiology and complicated pathogenesis. Moreover, the performances of hemodynamics, myocardial fibrosis, and cardiac hypertrophy at each stage vary, and the levels involved are diverse, ranging from cellular to molecular to genetic. Consequently, the pathogenesis of CHF can be elucidated from multiple directions. Therefore, it is necessary to use models with different characteristics for more comprehensive analyses. Additionally, the process of human CHF is often accompanied by other diseases, such as hypertension or diabetes, but this is rare in animal and cell models, so a combination of multiple technologies is required (Geng and Liu, 2014), which should be the focus of future research.

In our evaluation of experiments in CHF animals, the modeling method has a substantial role in the overall effect. According to our subgroup analysis, variability in modeling methods was associated with the levels of BNP, HR, HWI, ALD, LVSP, +dP/dt_max_, and −dP/dt_max_. The significance of the results of the above outcome indexes varied when subgroups were generated based on the modeling methods, sometimes from valid to invalid or the reverse, indicating a significant influence of the choice of modeling methods on the pooled results. More interestingly, we found that for the hemodynamics indicators (HR, LVSP, +dP/dt_max_, −dt/dt_max_), the DOX-inducement modeling method was more likely to achieve a valid positive result on the pooled effects of compatibility than AAC, indicating that research on the hemodynamic process of CHF should use the DOX-inducement model. For research on the ventricular remodeling index (e.g., HWI), TAC showed a significant overall effect of compatibility, whereas DOX-inducement and AAC did not, indicating that the TAC modeling method should be chosen. Furthermore, for research on the appropriate level of ALD to investigate how RAAS affects CHF, according to our subgroup analysis on ALD, the DOX-inducement method is recommended for establishing a CHF model if a positive outcome is expected. However, if the research focuses on the level of BNP, surgery methods would be more suitable. With the continuous development of experimental zoology, some models will more accurately reflect future clinical results and be more stable, easier to use, economic, and reproducible, all of which should promote a more comprehensive understanding of CHF in humans. The focus of the present research was mainly on how to choose a more reasonable and more appropriate model for a better exploration of the different aspects of CHF, which depends on the model–effect relationship, for eventual use in the design of pre-clinical trials.

Moreover, we investigated the time–effect relationship, which also has a critical role in clinical medicinal treatments. Subgroup analysis revealed that the difference in treatment duration was associated with the overall effects of HR, LVSP, +dP/dt_max_, and −dP/dt_max_, suggesting that duration had an influence on the ultimate results and that potential time–effect relationships should be explored. All things considered, these findings indicated that in pre-clinical studies of Fuzi compatibility effects in CHF animals, more attention should be given to the importance of establishing appropriate animal models and medication durations.

### 4.3 Limitations

The following limitations in this systematic review and meta-analysis were inevitable and should be considered carefully: 1) Neutral and negative results are normally less likely to be published in clinical trials, which increases the publication bias, thus exaggerating the effects of interventions. In our publication bias and small-study effect tests, the different degrees of asymmetry in the funnel plots and the statistical analysis results of Egger’s test both indicated that publication bias and small-study effects should not be ignored and that the efficacies of compatible interventions were probably exaggerated, so the positive results of the Fuzi compatibility effects in CHF animals should be cautiously interpreted. 2) In the quality assessment of the included studies, some were evaluated as of poor methodological quality for various reasons, such as the unclear illustration of random allocation, which may have led to selection bias. 3) In the meta-analysis, a few studies were not included for certain outcome measures because of insufficient or unavailable data even though positive results were mentioned, but those studies were still included in the systematic review.

## 5 Conclusion

This systematic review and meta-analysis aimed to evaluate the efficacy of Fuzi compatibility on CHF animals and identify potential synergistic compatibility mechanisms. The results showed that Fuzi compatibility has therapeutic effects against CHF animals superior to those of using Fuzi alone, and the potential synergistic compatibility mechanisms were associated with improvements in the hemodynamic process, increased activity of Na ^+^ -K ^+^ -ATPase, suppression of Ca^2+^ overload, regulation of the RAAS, inhibition of pro-inflammatory cytokines, alleviation of inflammation, improvements in ventricular remodeling and cardiac damage, influence on the metabolic process *in vivo*, and inhibition of cardiomyocyte apoptosis. We also found that the variations in modeling methods of CHF and medication duration influenced the pooled effects and thereby affected the stability of positive results, through which we concluded that there were possible model–effect and time-effect relationships. It should be clarified that factors, such as publication bias or other bias, the methodological quality of the included studies, and the small sample size effect can limit the accuracy and credibility of the positive results, which reminded us to dialectically treat the outcome of this paper. We hope more reasonably designed, fully reported, and high-quality pre-clinical animal experiments will be carried out so that more credible evidence can be provided for future clinical trials.
