# Supplementary material for: The synergistic compatibility mechanisms of fuzi against chronic heart failure in animals: A systematic review and meta-analysis
Source: Front Pharmacol. 2022 Sep 14;13:954253. doi: 10.3389/fphar.2022.954253 (PMC9515783; doi:10.3389/fphar.2022.954253)
Supplement: Supplementary file 1 [file Table5.pdf]

Table 5 Subgroup analysis according to HWI

Variables	Participants(n)	MD [95% CI]	P value (Significance tests)
MODEL of CHF			
drug(DOX)	57	-0.079 [-0.237, 0.078]	0.323
surgery(AAC)	70	-0.072 [-0.177, 0.034]	0.185
surgery(TAC)	13	-0.120 [-0.138, -0.102]	0.000
Duration			
<21days	13	-0.120 [-0.138, -0.102]	0.000
≥21days	127	-0.078 [-0.155, -0.002]	0.045
