# Supplementary material for: The synergistic compatibility mechanisms of fuzi against chronic heart failure in animals: A systematic review and meta-analysis
Source: Front Pharmacol. 2022 Sep 14;13:954253. doi: 10.3389/fphar.2022.954253 (PMC9515783; doi:10.3389/fphar.2022.954253)
Supplement: Supplementary file 2 [file Table4.pdf]

Table 4 Subgroup analysis according to HR

Variables	Participants(n)	MD [95% CI]	P value (Significance tests)
MODEL of CHF			
drug(DOX)	138	32.739 [6.400, 59.078]	0.015
surgery(AAC)	70	7.427 [-9.454, 24.309]	0.388
Duration			
<21days	138	32.739 [6.400, 59.078]	0.015
≥21days	70	7.427 [-9.454, 24.309]	0.388
