# Supplementary material for: The synergistic compatibility mechanisms of fuzi against chronic heart failure in animals: A systematic review and meta-analysis
Source: Front Pharmacol. 2022 Sep 14;13:954253. doi: 10.3389/fphar.2022.954253 (PMC9515783; doi:10.3389/fphar.2022.954253)
Supplement: Supplementary file 3 [file Table11.pdf]

ABBREVIATION

CHF	Chronic Heart Failure
TCM	Traditional Chinese Medicine
Fuzi	Radix *Aconiti Lateralis Preparata*
CNKI	China National Knowledge Infrastructure
CBM	China Biological Medicine Database
HF	Heart Failure
CVDs	Cardiac Vascular Diseases
DDAs	Diester-Diterpene Alkaloids
AC	Aconitine
MA	Mesaconitine
HA	Hypaconitine
SND	Sini Decoction
SFD	Shenfu Decoction
BNP	Brain Natriuretic Peptide
HR	Heart Rate
HWI	Heart Weight Index
ALD	Aldosterone
LVEDP	Left Ventricular End-Diastolic Pressure
LVSP	Left Ventricular Systolic Pressure
EF	Ejection Fraction
FS	Fractional Shortening

+dp/dtmax	maximum increase rate of left ventricular pressure
-dp/dtmax	maximum decline rate of left ventricular pressure
Ang II	Angiotensin II
ET-1	Endothelin 1
TNF-α	Tumor Necrosis Factor-α
CK	Creatine Kinase
LDH	Lactate Dehydrogenase
ATP	Adenosine Triphosphate
RCTs	Randomized Controlled Trails
WMD	Weighted Mean Difference
CI	Confidence Interval
Chi2	Chi-square
I2	I-square
DOX	Doxorubicin
TAC	Transverse Aortic Constriction
AAC	Abdominal Aorta Constriction
LAD	Left Anterior Descending Coronary Artery Ligation
HG	Higenamine
TA	Total Alkaloids
GA	Glycyrrhetinic Acid
[6]-GR	[6]-gingerol
TAG	Total Alkaloids and Total Gingerols
TAFS	Total Alkaloids and Total Flavones and Saponins

TAGFS	Total Alkaloids and Total Gingerols and Total Flavones and Saponins
LV	Left Ventricular
HE	Hematoxylin-Eosin
RAAS	Renin-Angiotensin-Aldosterone System
IL	Interleukin
Bcl-2	B-cell lymphoma-2
Bax	Bcl-2 associated X protein
Fas	Fatty acid synthetase
BAC	Benzoylaconine
BHA	Benzoylhypaconine
BMA	Benzoylmesaconine
ANP	Atrial Natriuretic Peptide
AHF	Acute Heart Failure
NT-ProBNP	N-terminal pro-Brain Natriuretic Peptide
