# Supplementary material for: The synergistic compatibility mechanisms of fuzi against chronic heart failure in animals: A systematic review and meta-analysis
Source: Front Pharmacol. 2022 Sep 14;13:954253. doi: 10.3389/fphar.2022.954253 (PMC9515783; doi:10.3389/fphar.2022.954253)
Supplement: Supplementary file 4 [file Table1.pdf]

Table 1 Characteristics of included studies

Study Year	n=(E, C)	Gender	Species	Weight(g)	Modeling Method	Compatibility	Single	Duration (days)	Outcome index
Xie GH 2021	8, 8	male female	Rat	180-220	surgery(LAD)	Red ginseng	Fuzi	7	1,3,16
Jia HH 2019	10, 9	male	Rat	180-220	drug(DOX)	Shan-zhu-yu	Fuzi	21	1,14
Fu N 2018	8, 8	male	Rat	170-210	surgery(TAC)	GA/liquiritin	HA	7	7,8,11
Wan JY 2017	7/8/9, 6	male	Rat	60-170	surgery(TAC)	GA/liquiritin	HA	7	1,7,8
Xu FL 2016	10, 9	male	Rat	190-210	drug(DOX)	Ginseng	AC	30	6,9,10,13
Jin Z 2015	8, 8	male female	Rat	180-220	surgery(AAC)	Shan-zhu-yu	Fuzi	42	1,5,6,9,10,13
Miao P 2015	10/10/10, 9	male	Rat	180-220	drug(DOX)	SND/Gancao/Dried ginger	Fuzi	28	1,3,4,11
Liang T 2014	10/10, 10/10	male female	Rat	200-220	drug(DOX)	Dried ginger	AC	16	12,14
Zhai JY 2013	7/7/7, 7	male	Rat	200-250	drug(DOX)	SND/Gancao/ Dried ginger	Fuzi	12	2,5,6,9,10
Yang HR 2013	8/8/8/8/8 /8, 8/8	NR	Rat	180-220	drug(DOX)	SND/Gancao/ Dried ginger	Fuzi	14	2,5,6,9,10

Xu L 2015	7/7/8/6/7, 7	male	Rat	250-300	surgery(AAC)	Red ginseng	Fuzi	42	1,2,3,4,5,6,9,10
Peng H 2018	6/6/6/6/6/6/6/6, 6/6	male	Rat	180-220	surgery(LAD)	Fuling	Hei-shun-pian	14	11,15,16
Yang L 2019	7, 6	male	Rat	180-220	surgery(LAD)	Red ginseng	Fuzi	21	1,2,3,5,6,9,10,13
Wu JT 2018	9, 7	male	Rat	180-220	surgery(LAD)	Ginseng	Fuzi	56	1,7,8
Yan P 2020	10, 10	male	Mouse	20-24	surgery(TAC)	Gancao	Fuzi	56	2,3,5,7,8,9,10,13
Wen JX_A 2020	6, 6	male	Rat	180-220	drug(DOX)	Dried ginger	Fuzi	21	4,5,6,9,10,11,12
Wen JX_B 2020	NR	male	Rat	180-220	drug(DOX)	[6]GR	HG	7	4,5,6,9,10,11,12
Wen JX 2019	10/10/10, 10/10/10	male	Rat	160-200	drug(DOX)	Dried ginger	Fuzi	21	1,5,6,9,10,15,16
Sun FJ 2019	15, 15	male	Rat	230-250	surgery(AAC)	Banxia	Fuzi	42	1,2,7
Sun FJ 2018	15, 15	male	Rat	240-260	drug(DOX)	Banxia	Fuzi	14	1,2,7
Wang LQ 2016	7, 8	male	Rat	160-170	surgery(TAC)	GA	HA	7	1,3,7,8
Chen S 2014	10/10/10/10, 10	NR	Mouse	NR	drug(DOX)	TAFS/TAGFS/TAG/SND	TA	NR	15,16

Miao P 2016	10/10/10, 9	male	Rat	180-220	drug(DOX)	SND/Gancao/Dried ginger	Fuzi	28	17
Ni LL 2022	6, 6	NR	Rat	180-200	drug(DOX)	Gancao	Fuzi	28	1,4,11,17

NR: not reported; Outcome index: 1. BNP; 2. HR; 3. HWI; 4. ALD; 5. LVEDP; 6. LVSP; 7. EF; 8. FS; 9. +dp/dtmax; 10. −dp/dtmax; 11. Ang II; 12. ET-1; 13. TNF-α; 14. Na^+-K^+-ATPase; 15. CK; 16. LDH; 17. ATP
