# Supplementary material for: The synergistic compatibility mechanisms of fuzi against chronic heart failure in animals: A systematic review and meta-analysis
Source: Front Pharmacol. 2022 Sep 14;13:954253. doi: 10.3389/fphar.2022.954253 (PMC9515783; doi:10.3389/fphar.2022.954253)
Supplement: Supplementary file 5 [file Table2.pdf]

Table 2 Quality assessment of the included studies

Study Year	I	II	III	IV	V	VI	VII	VIII	IX	X
Xie GH 2021	U	L	U	U	U	U	U	L	L	L
Jia HH 2019	U	L	U	U	U	U	U	L	L	L
Fu N 2018	L	L	U	U	U	U	U	L	L	L
Wan JY 2017	U	L	U	U	U	U	U	L	L	L
Miao P 2016	L	L	U	U	U	U	U	L	L	L
Xu FL 2016	U	L	U	U	U	U	U	L	L	U
Jin Z 2015	U	L	U	U	U	U	U	L	L	L
Miao P 2015	L	L	U	U	U	U	U	L	L	L
Liang T 2014	U	L	U	U	U	U	U	L	L	L
Zhai JY 2013	U	L	U	U	U	U	U	L	L	L
Yang HR 2013	U	L	U	U	U	U	U	L	L	U
Xu L 2015	U	L	U	U	U	U	U	L	L	U
Peng H 2018	U	L	U	U	U	U	U	L	L	L
Yang L 2019	U	L	U	U	U	U	U	L	L	U
Wu JT 2018	U	L	U	U	U	U	U	L	L	U
Yan P 2020	U	L	U	U	U	U	U	L	L	L

Wen JX_A 2020	U	L	U	U	U	U	U	L	L	L
Wen JX_B 2020	U	L	U	U	U	U	U	L	L	L
Wen JX 2019	U	L	U	U	U	U	U	L	L	L
Sun FJ 2019	U	L	U	U	U	U	U	L	L	L
Sun FJ 2018	U	L	U	U	U	U	U	L	L	L
Wang LQ 2016	U	L	U	U	U	U	U	L	L	L
Chen S 2014	U	L	U	U	U	U	U	L	L	L
Ni LL 2022	U	L	U	U	U	U	U	L	L	L

Selection bias: **I**-sequence generation, **II**-baseline characteristics, **III**-allocation concealment; ***Performance bias***: **IV**-random housing, **V**-blinding of caregivers and/or investigators; ***Detection bias***: **VI**-random outcome assessment, **VII**-blinding of the outcome assessor; ***Attribution bias***: **VIII**-incomplete outcome data; ***Reporting bias***: **IX**-selective outcome reporting; **Other sources of bias**: **X**-funding; H: high risk of bias, L: low risk of bias, U: unclear risk of bias.
