# Supplementary material for: The synergistic compatibility mechanisms of fuzi against chronic heart failure in animals: A systematic review and meta-analysis
Source: Front Pharmacol. 2022 Sep 14;13:954253. doi: 10.3389/fphar.2022.954253 (PMC9515783; doi:10.3389/fphar.2022.954253)
Supplement: Supplementary file 6 [file Table9.pdf]

Table 9 Subgroup analysis according to +dp/dtmax

Variables	Participants(n)	MD [95% CI]	P value (Significance tests)
MODEL of CHF			
drug(DOX)	169	1483.571 [577.808, 2389.333]	0.001
surgery(AAC)	86	-55.958 [-717.239, 605.322]	0.868
Duration			
<21days	138	1736.817 [660.395, 2813.238]	0.002
≥21days	117	231.215 [-543.364, 1005.794]	0.559
