# Supplementary material for: The synergistic compatibility mechanisms of fuzi against chronic heart failure in animals: A systematic review and meta-analysis
Source: Front Pharmacol. 2022 Sep 14;13:954253. doi: 10.3389/fphar.2022.954253 (PMC9515783; doi:10.3389/fphar.2022.954253)
Supplement: Supplementary file 7 [file Table6.pdf]

Table 6 Subgroup analysis according to ALD

Variables	Participants(n)	MD [95% CI]	P value (Significance tests)
MODEL of CHF^			
drug(DOX)	48	-168.021 [-260.951, -75.092]	0.000
surgery(AAC)	70	-1.039 [-3.677, 1.598]	0.440

^: source of significant heterogeneity.
