# Supplementary material for: The synergistic compatibility mechanisms of fuzi against chronic heart failure in animals: A systematic review and meta-analysis
Source: Front Pharmacol. 2022 Sep 14;13:954253. doi: 10.3389/fphar.2022.954253 (PMC9515783; doi:10.3389/fphar.2022.954253)
Supplement: Supplementary file 8 [file Table3.pdf]

Table 3 Subgroup analysis for BNP

Variables	Participants(n)	MD [95% CI]	P value (Significance tests)
MODEL of CHF			
drug(DOX)	56	-4.528 [-18.408, 9.352]	0.523
surgery(AAC)	88	-57.419 [-88.458, -26.381]	0.000
surgery(TAC)	57	-807.696 [-1.4e+03, -171.678]	0.013
surgery(LAD)	19	-96.800 [-128.677, -64.923]	0.000
Duration			
<21days	57	-807.696 [-1.4e+03, -171.678]	0.013
≥21days	163	-41.656 [-67.957, -15.354]	0.002
