# Supplementary material for: The synergistic compatibility mechanisms of fuzi against chronic heart failure in animals: A systematic review and meta-analysis
Source: Front Pharmacol. 2022 Sep 14;13:954253. doi: 10.3389/fphar.2022.954253 (PMC9515783; doi:10.3389/fphar.2022.954253)
Supplement: Supplementary file 9 [file Table7.pdf]

Table 7 Subgroup analysis according to LVEDP

Variables	Participants(n)	MD [95% CI]	P value (Significance tests)
MODEL of CHF			
drug(DOX)	150	-1.426 [-6.025, 3.174]	0.332
surgery(AAC)	86	1.758 [-1.795, 5.310]	0.543
Duration			
<21days	138	-0.622 [-1.829, 0.584]	0.312
≥21days	98	0.009 [-5.650, 5.668]	0.998
