# Supplementary material for: The synergistic compatibility mechanisms of fuzi against chronic heart failure in animals: A systematic review and meta-analysis
Source: Front Pharmacol. 2022 Sep 14;13:954253. doi: 10.3389/fphar.2022.954253 (PMC9515783; doi:10.3389/fphar.2022.954253)
Supplement: Supplementary file 10 [file Table8.pdf]

Table 8 Subgroup analysis according to LVSP

Variables	Participants(n)	MD [95% CI]	P value (Significance tests)
MODEL of CHF^			
drug(DOX)	159	12.603 [6.400, 18.807]	0.000
surgery(AAC)	82	-3.404 [-16.082, 9.275]	0.599
Duration			
<21days	138	12.494 [5.341, 19.647]	0.001
≥21days	113	3.999 [-5.322, 13.321]	0.400
