# Supplementary material for: The synergistic compatibility mechanisms of fuzi against chronic heart failure in animals: A systematic review and meta-analysis
Source: Front Pharmacol. 2022 Sep 14;13:954253. doi: 10.3389/fphar.2022.954253 (PMC9515783; doi:10.3389/fphar.2022.954253)
Supplement: Supplementary file 11 [file Image1.pdf]

APPENDIX 1 *Search Strategy*

PubMed: 13 results

#1 "heart failure"[MeSH Terms]

#2 "heart failure"[MeSH Terms] OR "cardiac failure"[Title/Abstract] OR "heart decompensation"[Title/Abstract] OR "decompensation heart"[Title/Abstract] OR "heart failure right sided"[Title/Abstract] OR "heart failure right sided"[Title/Abstract] OR "right sided heart failure"[Title/Abstract] OR "right sided heart failure"[Title/Abstract] OR "myocardial failure"[Title/Abstract] OR "congestive heart failure"[Title/Abstract] OR "heart failure congestive"[Title/Abstract] OR "heart failure left sided"[Title/Abstract] OR "heart failure left sided"[Title/Abstract] OR "left sided heart failure"[Title/Abstract] OR "left sided heart failure"[Title/Abstract]

#3 "aconitum"[MeSH Terms]

#4 "Aconitum"[MeSH Terms] OR (("plant roots"[MeSH Terms] OR ("plant"[All Fields] AND "roots"[All Fields]) OR "plant roots"[All Fields] OR "Radix"[All Fields] OR "radixes"[All Fields]) AND "Aconitus"[Title/Abstract]) OR "Aconitums"[Title/Abstract] OR "aconitum napellus"[Title/Abstract] OR "Monkshood"[Title/Abstract] OR "Monkshoods"[Title/Abstract] OR "Aconite"[Title/Abstract] OR "Aconites"[Title/Abstract] OR "radix aconiti"[Title/Abstract] OR "aconiti radix"[Title/Abstract] OR ("Aconitus"[All Fields] AND "Radix"[Title/Abstract]) OR (("plant roots"[MeSH Terms] OR ("plant"[All Fields] AND "roots"[All Fields]) OR "plant roots"[All Fields] OR "Radix"[All Fields] OR "radixes"[All Fields]) AND "Aconitus"[Title/Abstract])

#5 "rats"[MeSH Terms]

#6 "mice"[MeSH Terms]

#7 "Rats"[MeSH Terms] OR "Mice"[MeSH Terms] OR "Rat"[Title/Abstract] OR "Rattus"[Title/Abstract] OR "rattus norvegicus"[Title/Abstract] OR "rats norway"[Title/Abstract] OR "rats laboratory"[Title/Abstract] OR "laboratory rat"[Title/Abstract] OR "laboratory rats"[Title/Abstract] OR "rat laboratory"[Title/Abstract] OR "Mus"[Title/Abstract] OR "Mouse"[Title/Abstract] OR "mus domesticus"[Title/Abstract] OR "mus musculus domesticus"[Title/Abstract] OR "domesticus mus musculus"[Title/Abstract] OR "mus musculus"[Title/Abstract] OR "mice house"[Title/Abstract] OR "house mice"[Title/Abstract] OR "mouse house"[Title/Abstract] OR "house mouse"[Title/Abstract] OR "mouse swiss"[Title/Abstract] OR "swiss mouse"[Title/Abstract] OR "swiss mice"[Title/Abstract] OR "mice swiss"[Title/Abstract] OR "mice laboratory"[Title/Abstract] OR "laboratory

mice"[Title/Abstract] OR "mouse laboratory"[Title/Abstract] OR "laboratory mouse"[Title/Abstract]

#8 #2 AND #4 AND #7

The Cochrane Library: 1 result

#1 MeSH descriptor: [Heart Failure] explode all trees

#2 (HF):ti,ab,kw OR (Right Sided Heart Failure):ti,ab,kw OR (Heart Failure, Right Sided):ti,ab,kw OR (Heart Decompensation):ti,ab,kw OR (Heart Failure, Left Sided):ti,ab,kw OR (Left Sided Heart Failure):ti,ab,kw OR (Cardiac Failure):ti,ab,kw OR (Congestive Heart Failure):ti,ab,kw OR (Myocardial Failure):ti,ab,kw

#3 #1 OR #2

#4 MeSH descriptor: [Aconitum] explode all trees

#5 (Aconitum):ti,ab,kw OR (Aconiti, Radix):ti,ab,kw OR (Radix Aconiti):ti,ab,kw OR (Radix Aconitus):ti,ab,kw OR (Aconites):ti,ab,kw OR (Aconitus, Radix):ti,ab,kw OR (Aconite):ti,ab,kw OR (Monkshoods):ti,ab,kw OR (Monkshood):ti,ab,kw OR (Aconitum napellus):ti,ab,kw

#6 #4 OR #5

#7 MeSH descriptor: [Rats] explode all trees

#8 MeSH descriptor: [Mice] explode all trees

#9 (Rattus):ti,ab,kw OR (Rattus norvegicus):ti,ab,kw OR (Rat):ti,ab,kw OR (Rats, Norway):ti,ab,kw OR (Laboratory Rat):ti,ab,kw OR (domesticus, Mus musculus):ti,ab,kw OR (Mus domesticus):ti,ab,kw OR (Mus musculus domesticus):ti,ab,kw OR (Mouse):ti,ab,kw OR (Mus):ti,ab,kw OR (Laboratory Mouse):ti,ab,kw OR (Laboratory Mice):ti,ab,kw OR (House Mouse):ti,ab,kw OR (House Mice):ti,ab,kw OR (Mice, Swiss):ti,ab,kw OR (Mouse, Swiss):ti,ab,kw

#10 #7 OR #8 OR #9

#11 #3 AND #6 AND #10

Web of Science: 30 results
TOPIC: ("Chronic heart failure" OR heart failure OR cardiac failure OR myocardial failure) *AND* TOPIC: (aconitum* OR aconite*) *AND* TOPIC: (rat* OR mice)

Embase: 26 results

#1. 'heart failure'/exp OR 'heart failure'

#2. aconitum

#3. 'rat'

#4. 'mouse'

#5. #3 OR #4

#6. #1 AND #2 AND #5

CINAHL Complete: 9 results

Boolean/Phrase:
Heart failure AND Aconitum AND (Rat OR Mice)

Expanders:
Apply related words

CNKI: 58 results
（主题=附子 ）AND（摘要=配伍 + 减毒增效 + 增效 + 减毒）AND（摘要=大鼠 + 小鼠）AND（摘要=慢性心力衰竭 + 心力衰竭 + 慢性心衰 + 心衰）

Wangfang Data Information Site: 41 results
主题:(附子）and 摘要:(配伍 or 减毒增效 or 增效 or 减毒）and 摘要:(大鼠 or 小鼠）and 摘要:(慢性心力衰竭 or 慢性心衰 or 心力衰竭 or 心衰）

VIP Information Database: 20 results
(((题名或关键词=附子 AND (((文摘=配伍 OR 文摘=减毒增效) OR 文摘=增效) OR 文摘=减毒)) AND (文摘=大鼠 OR 文摘=小鼠)) AND (((文摘=慢性心力衰竭 OR 文摘=心力衰竭) OR 文摘=慢性心衰) OR 文摘=心衰))

CBM：28 results

″附子″[常用字段:智能] AND(″配伍″[常用字段:智能] OR ″减毒增效″[常用字段:智能] OR ″减毒″[常用字段:智能] OR ″增效″[常用字段:智能]) AND(″大鼠″[常用字段:智能] OR ″小鼠″[常用字段:智能]) AND(″慢性心力衰竭″[常用字段:智能] OR ″心力衰竭″[常用字段:智能] OR ″慢性心衰″[常用字段:智能] OR ″心衰″[常用字段:智能])

APPENDIX 2 *Sensitivity Analysis*

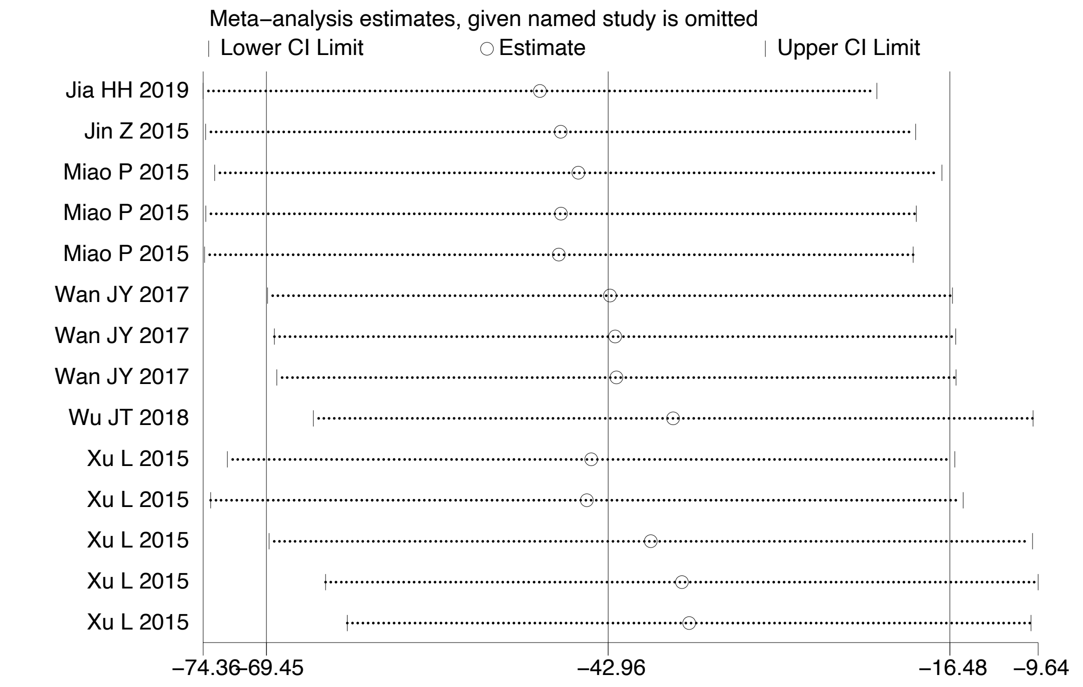

Figure 11 *Sensitivity analysis of BNP*

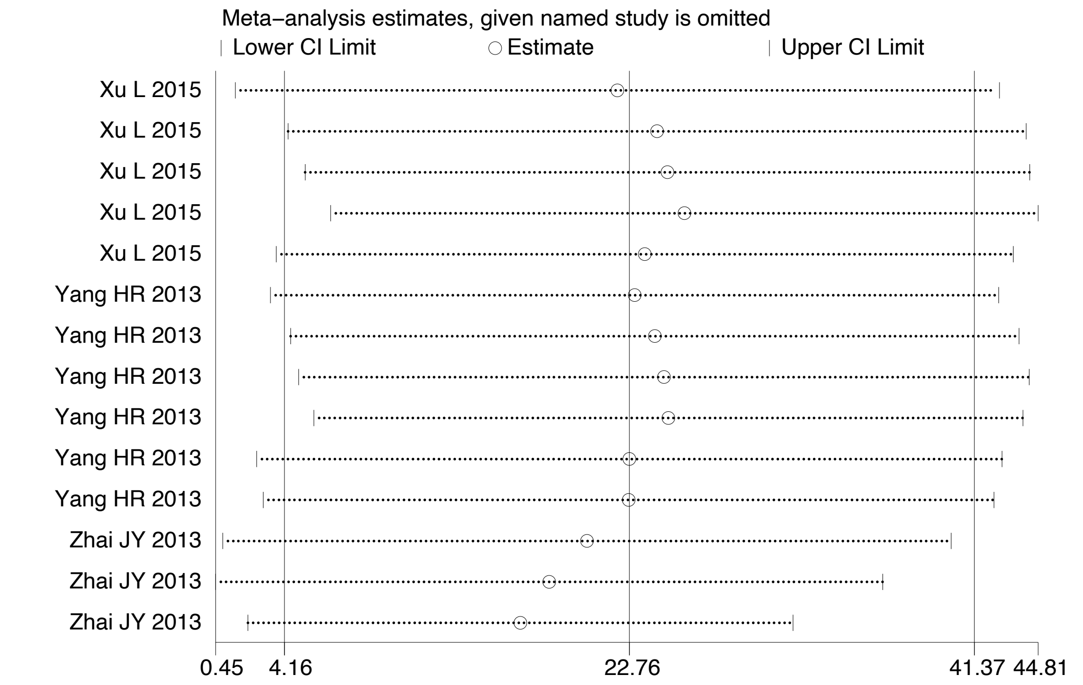

Figure 12 *Sensitivity analysis of HR*

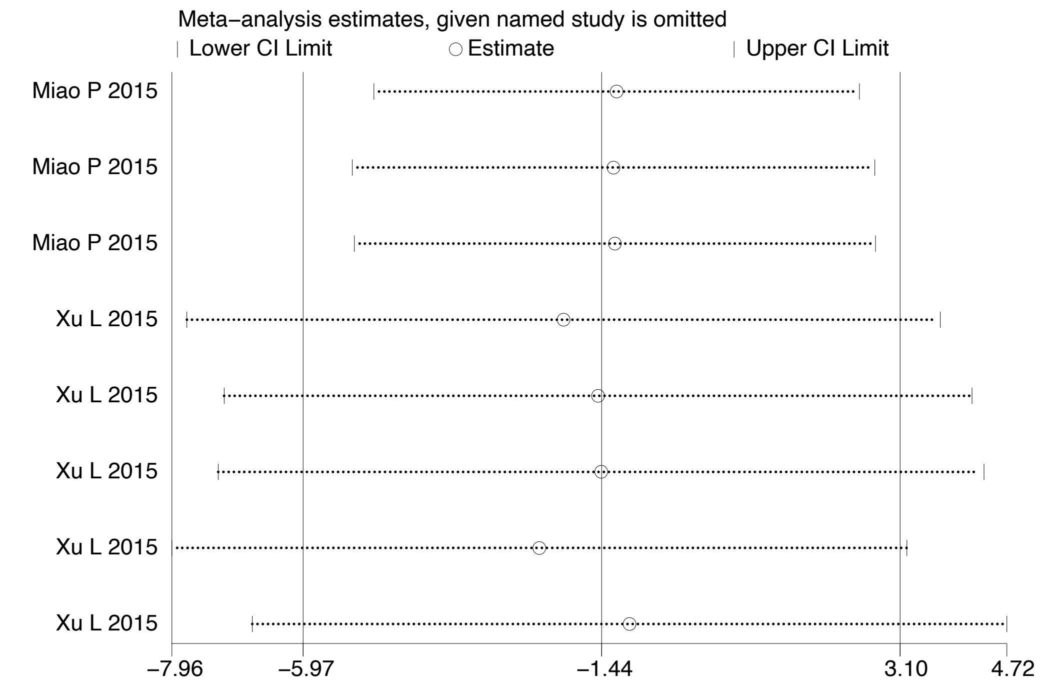

Figure 13 *Sensitivity analysis of ALD*

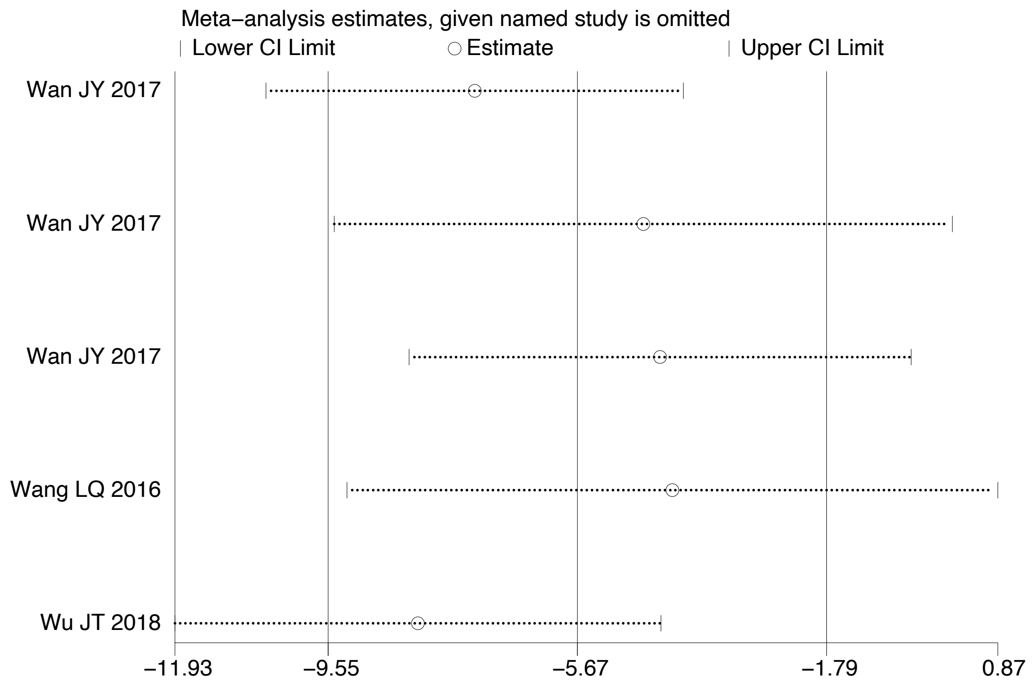

Figure 14 *Sensitivity analysis of EF*

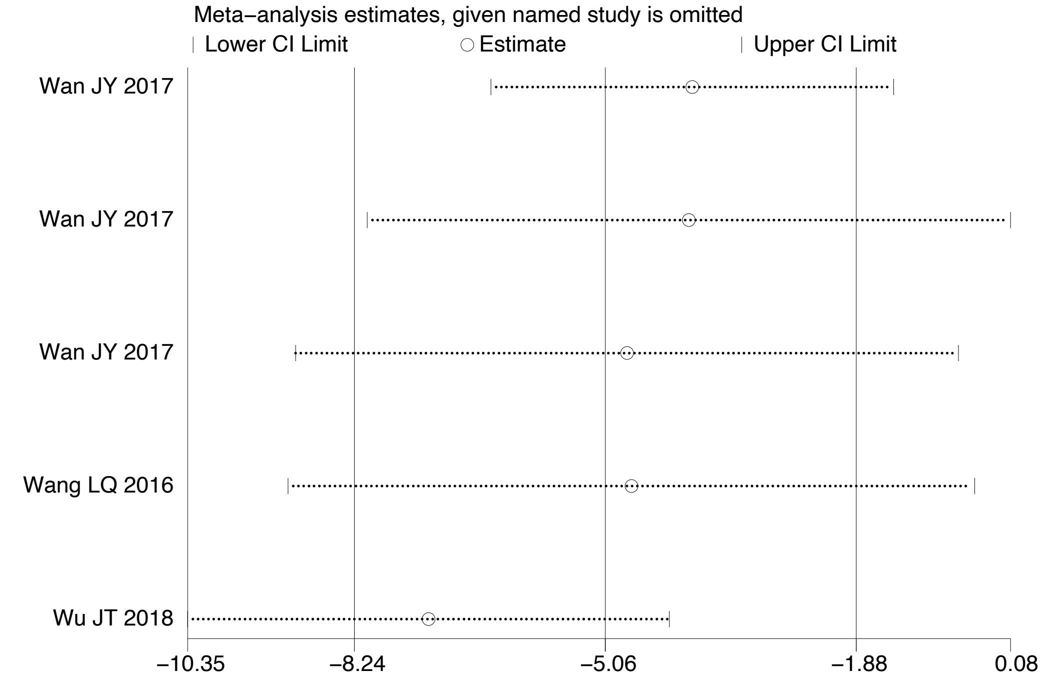

Figure 15 *Sensitivity analysis of FS*

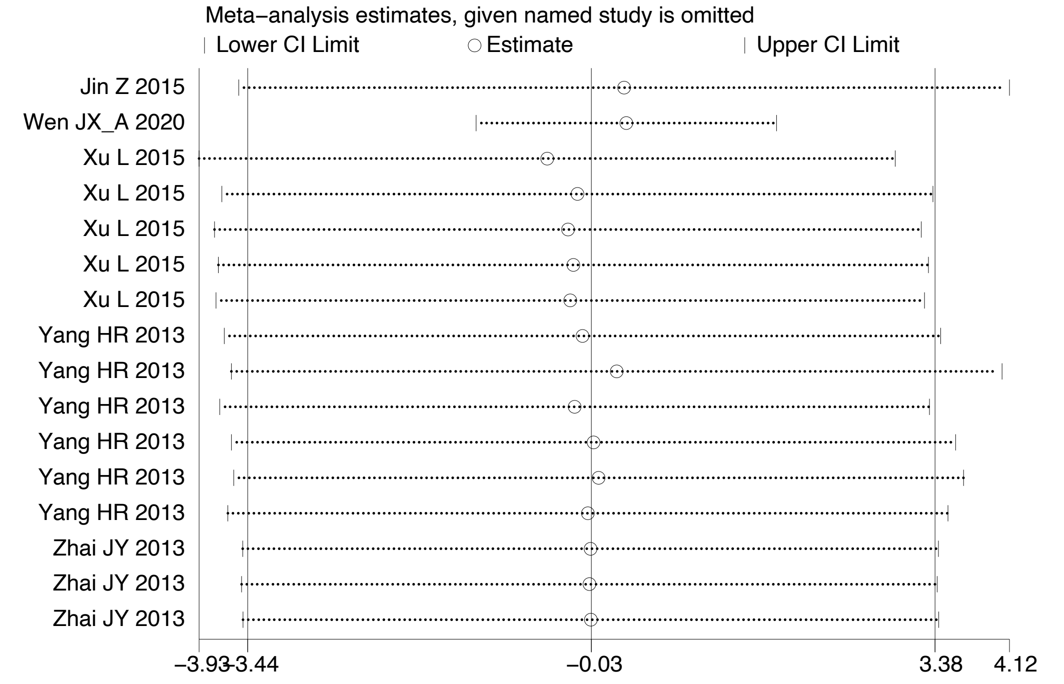

Figure 16 *Sensitivity analysis of LVEDP*

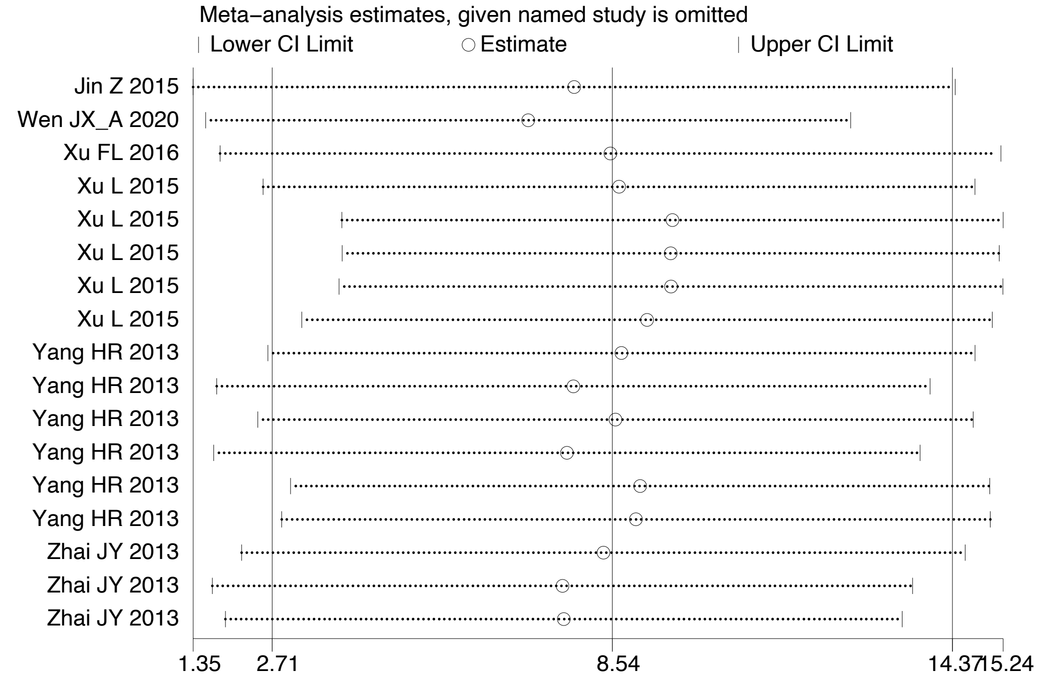

Figure 17 *Sensitivity analysis of LVSP*

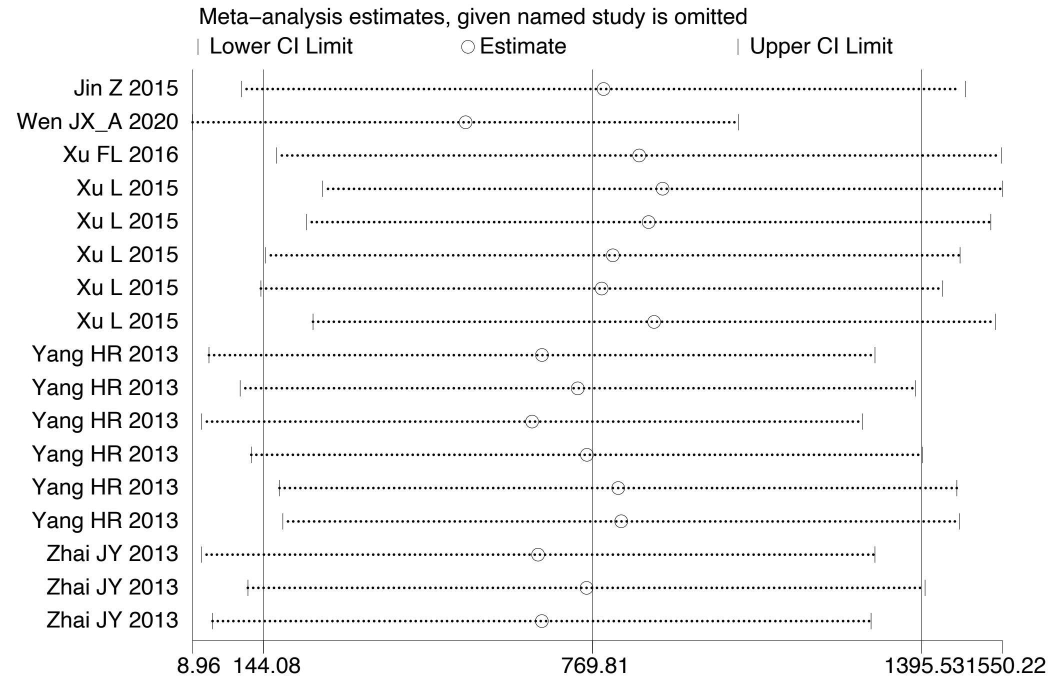

Figure 18 *Sensitivity analysis of +dp/dtmax*

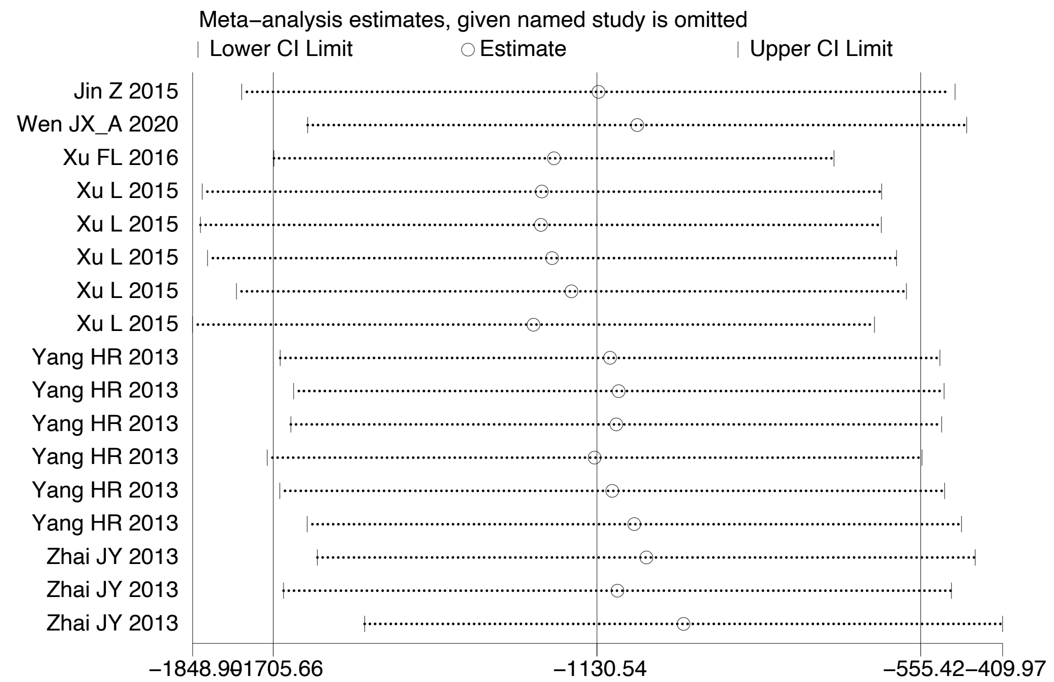

Figure 19 *Sensitivity analysis of -dp/dtmax*

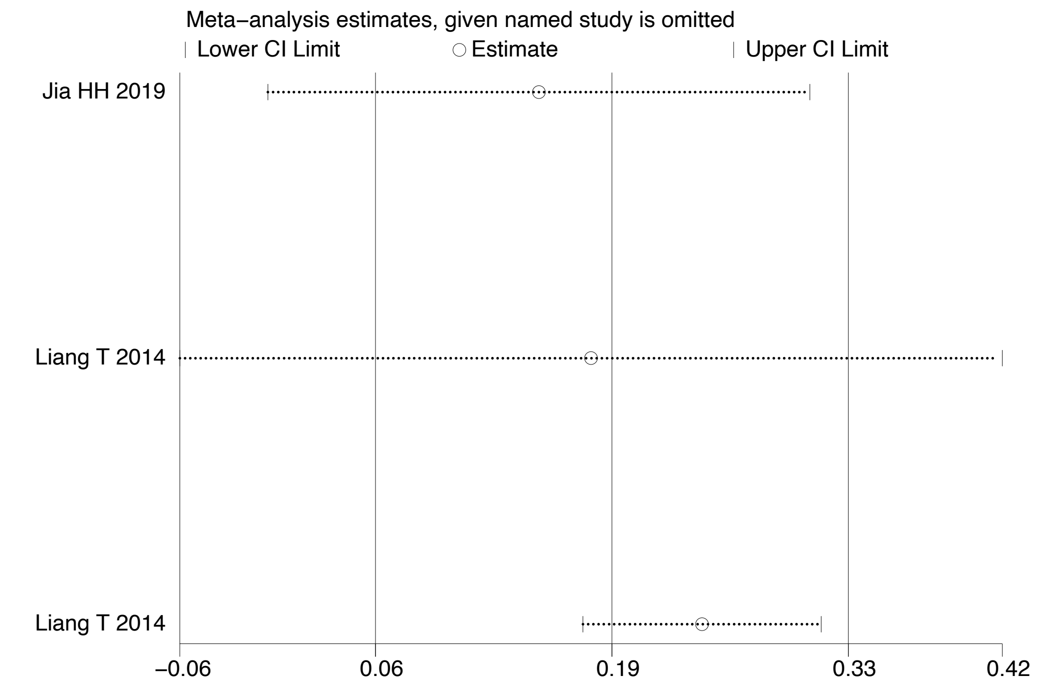

Figure 20 *Sensitivity analysis of* Na^{+}*-*K^{+}*-ATPase*

APPENDIX 3 *Publication Bias Assessment*

1. Funnel Plots

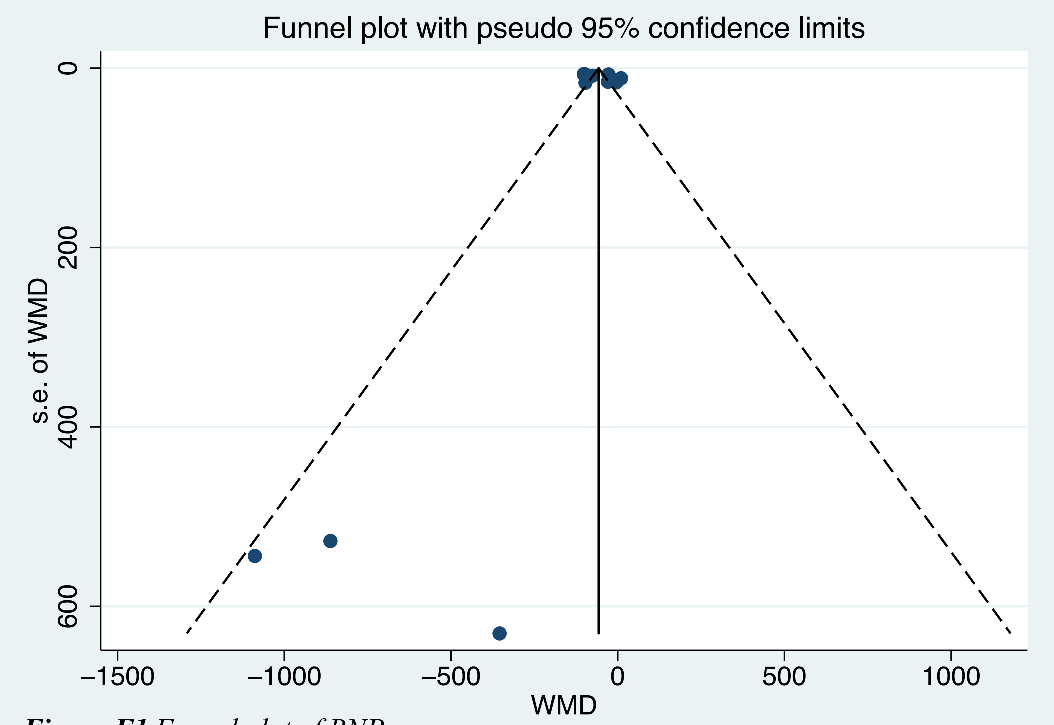

Figure F1 *Funnel plot of BNP*

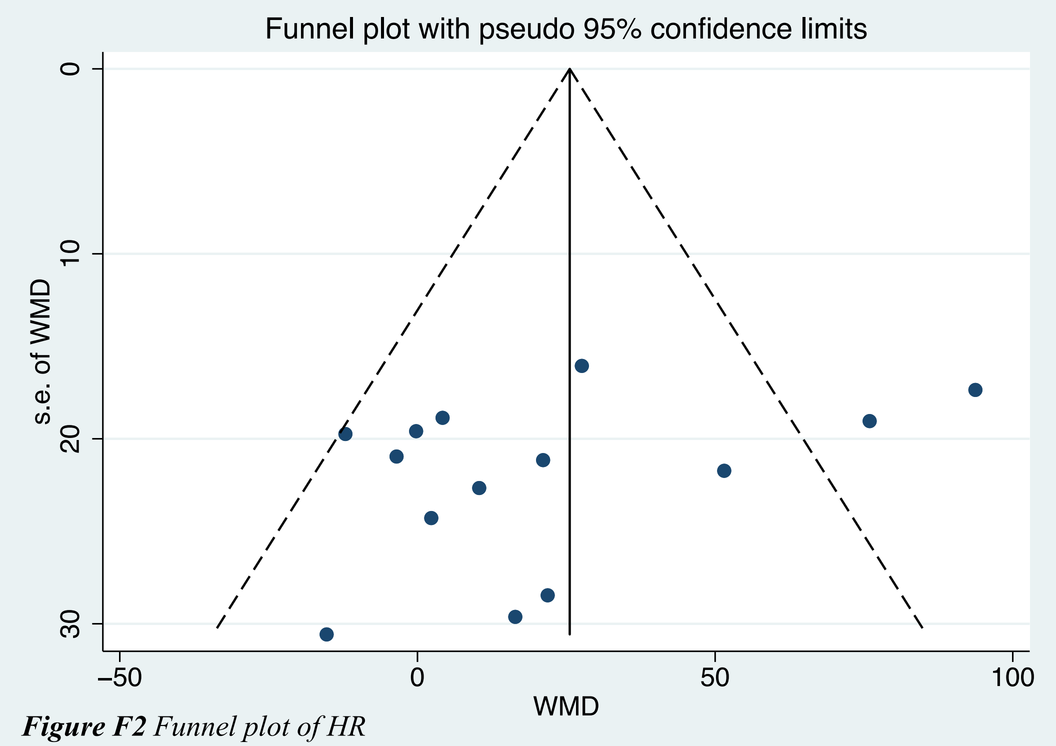

Figure F2 *Funnel plot of HR*

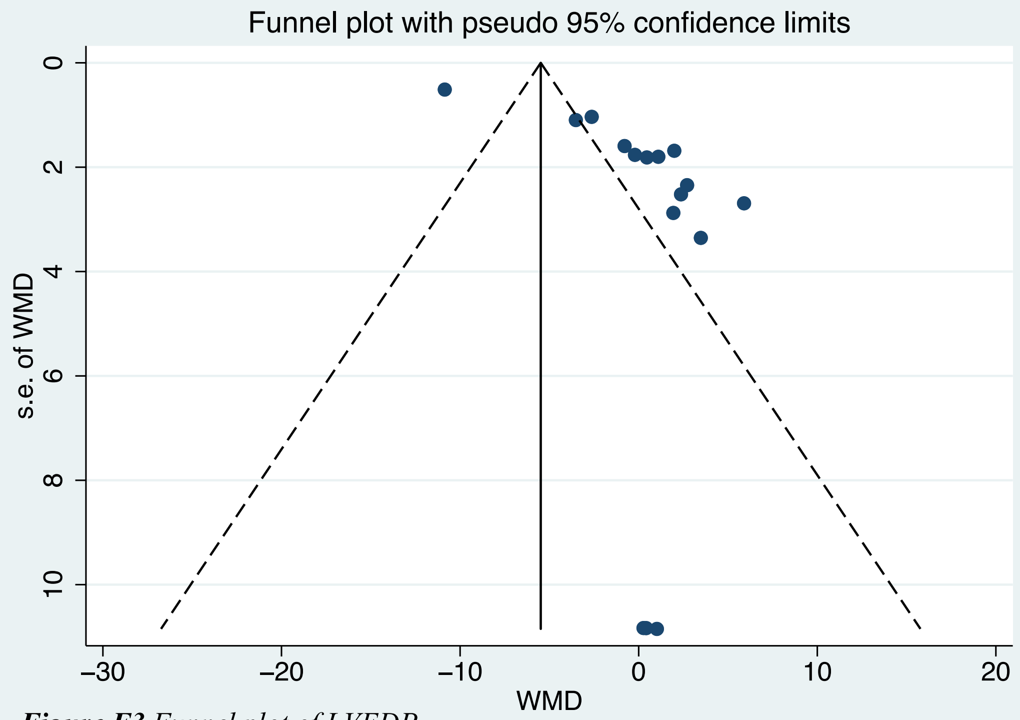

Figure F3 *Funnel plot of LVEDP*

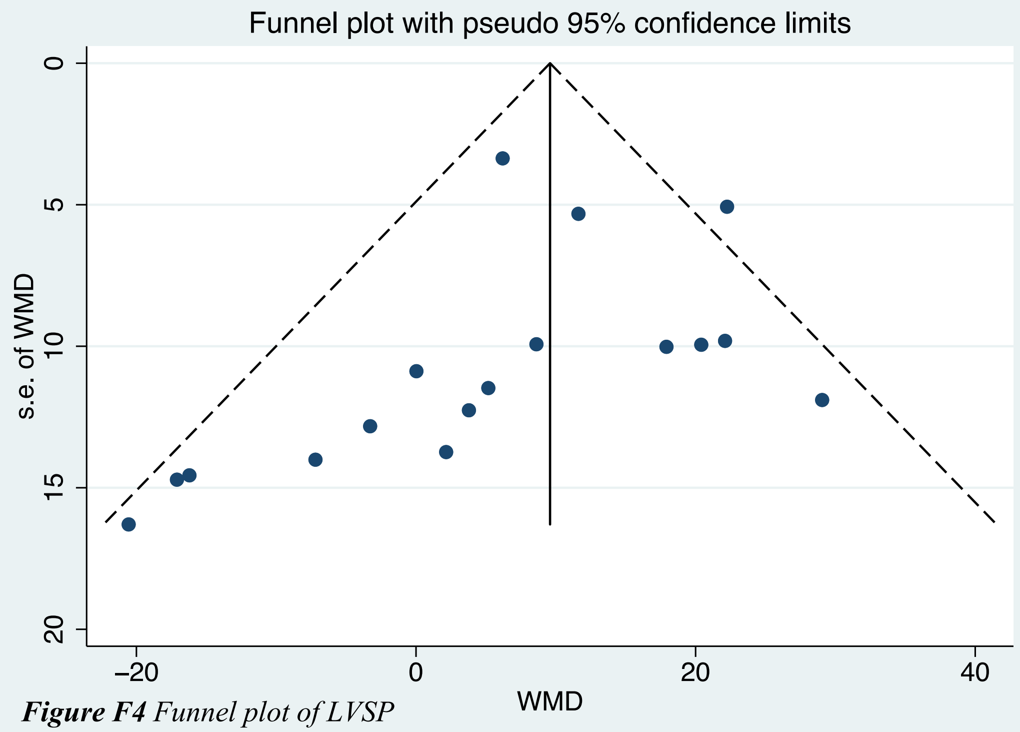

Figure F4 *Funnel plot of LVSP*

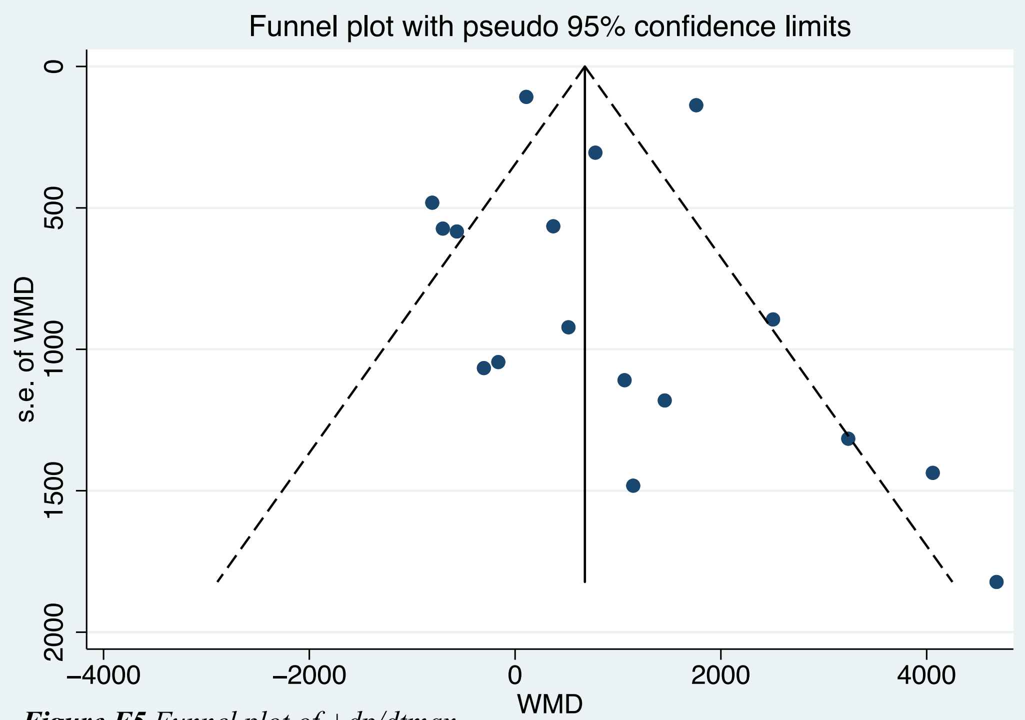

Figure F5 *Funnel plot of +dp/dtmax*

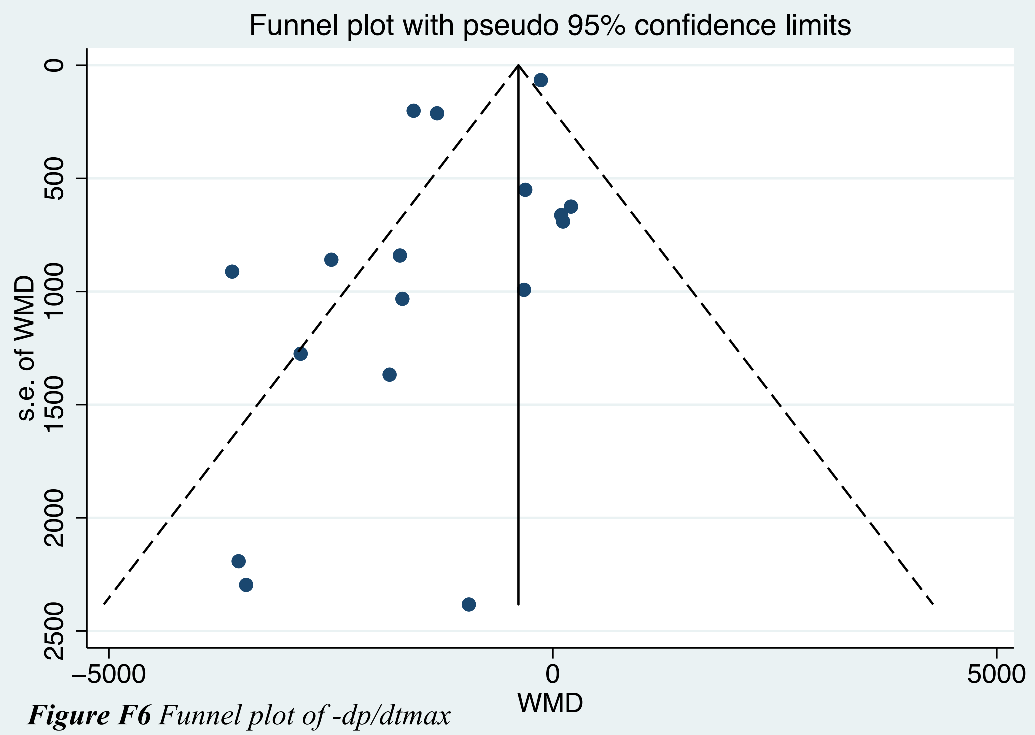

Figure F6 *Funnel plot of -dp/dtmax*

2. Egger's Test Results

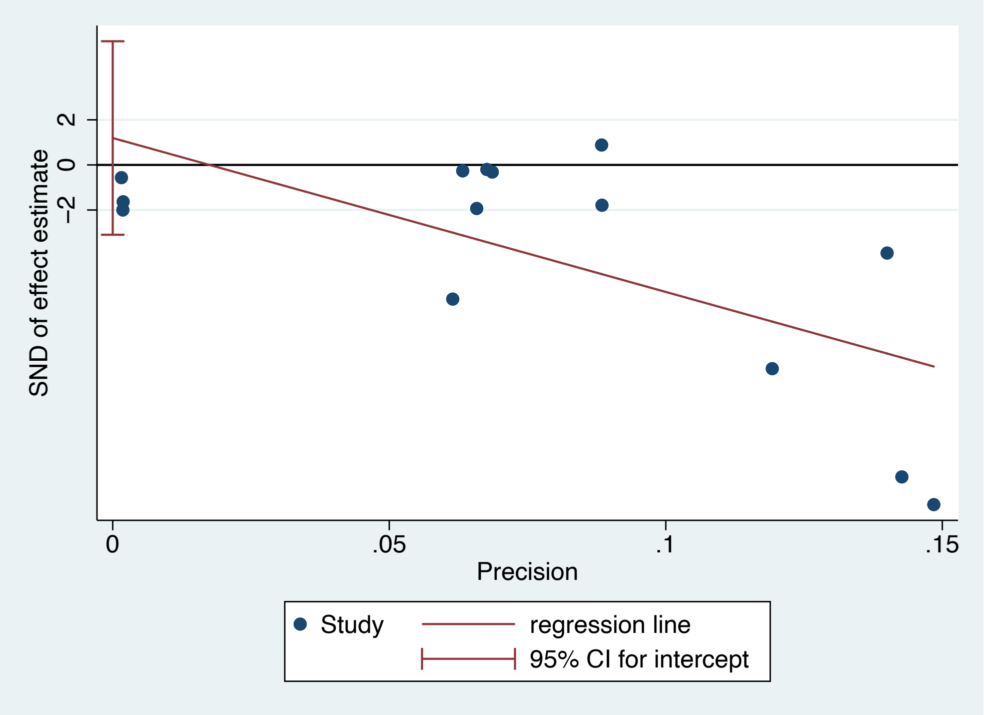

Figure E1 *Egger's test of BNP*
Bias: 1.19, P = 0.556, 95%CI [-3.10, 5.49]

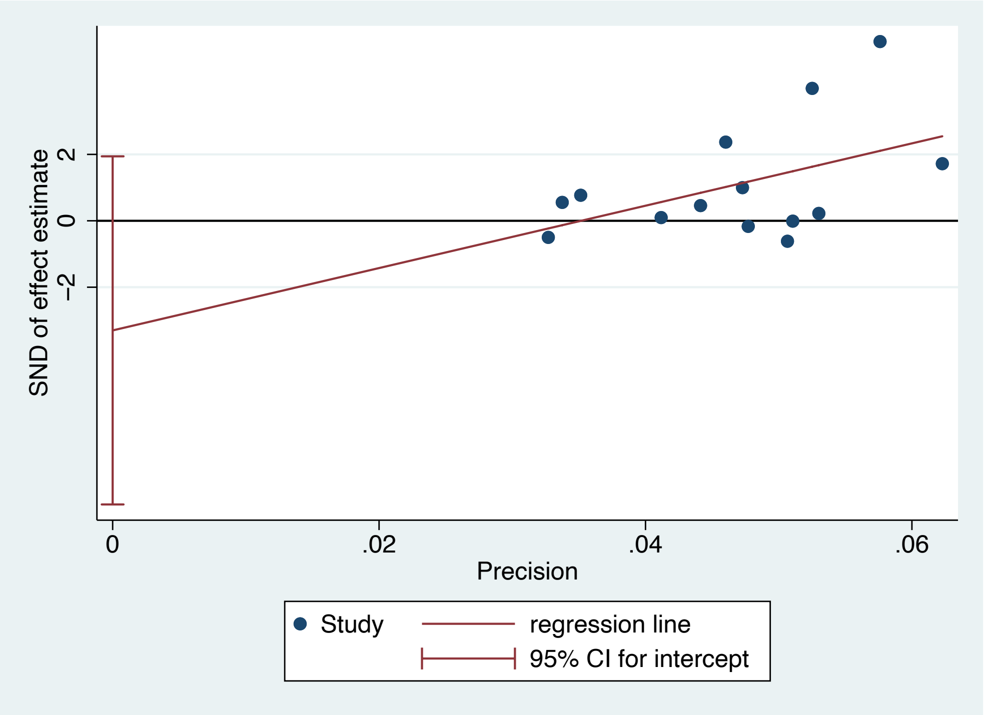

Figure E2 *Egger's test of HR*
Bias: -3.30, P = 0.195, 95%CI [-8.54, 1.94]

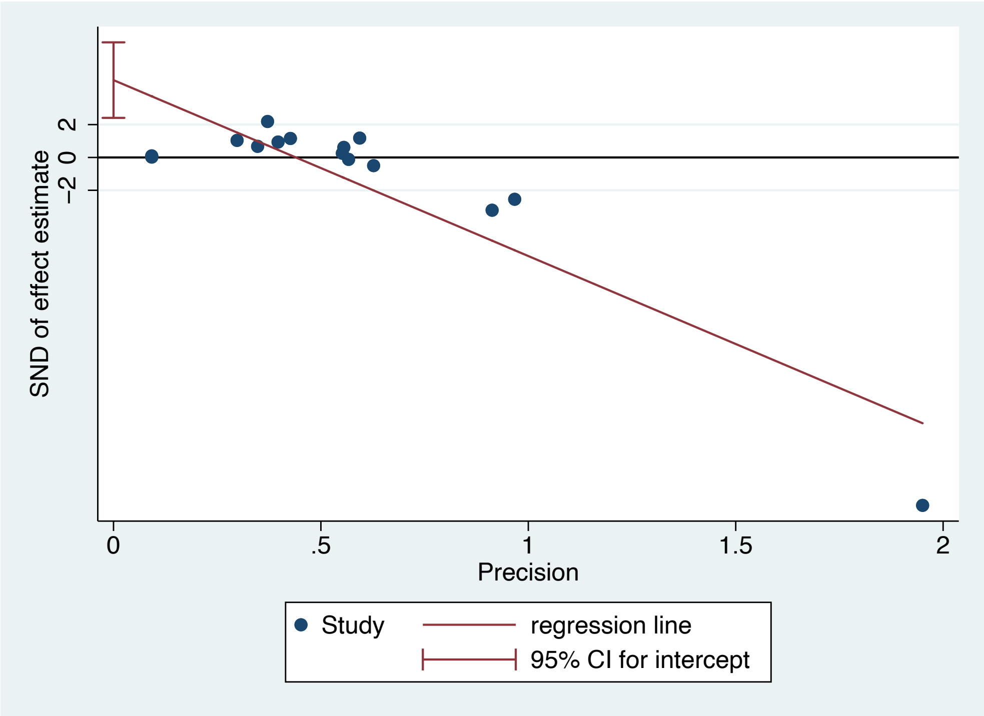

Figure E3 *Egger's test of LVEDP*
Bias: 4.71, P = 0.001, 95%CI [2.41, 7.01]

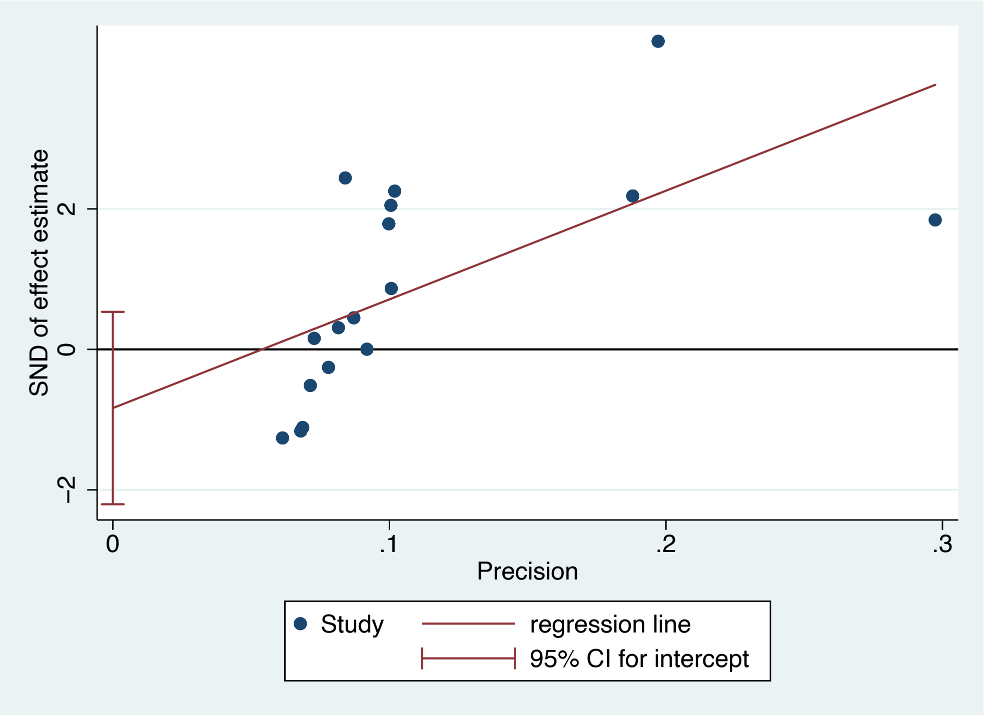

Figure E4 *Egger's test of LVSP*
Bias: -0.84, P = 0.213, 95%CI [-2.21, 0.53]

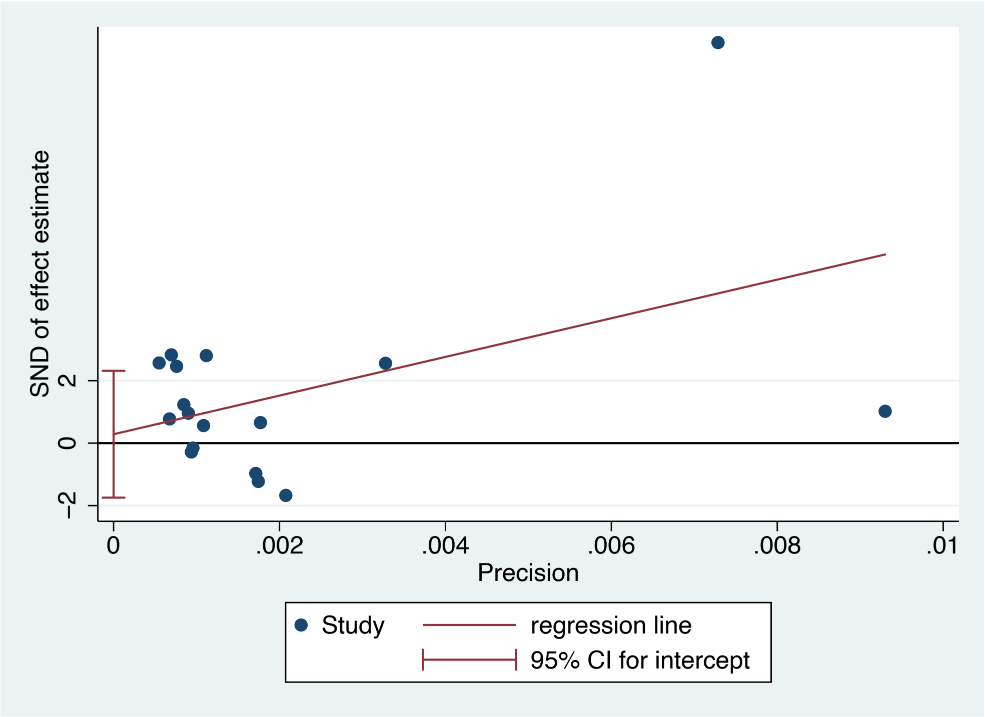

Figure E5 *Egger's test of +dp/dtmax*
Bias: 0.29, P = 0.769, 95%CI [-1.75, 2.32]

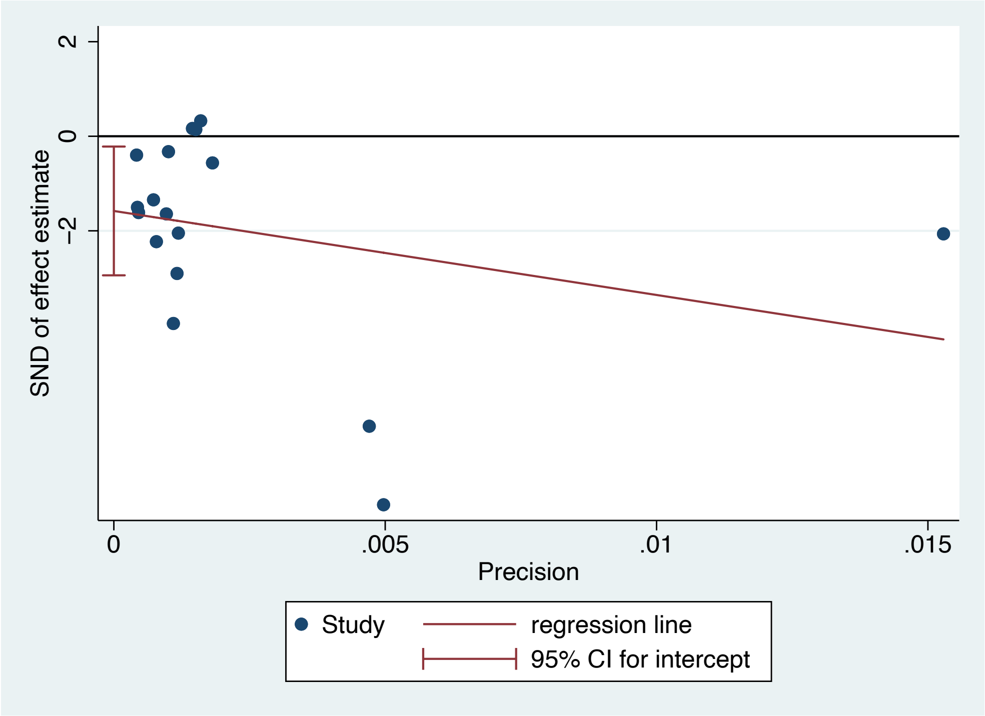

Figure E6 *Egger's test of -dp/dtmax*
Bias: -1.58, P = 0.026, 95%CI [-2.94, -0.22]
