# Supplementary material for: The synergistic compatibility mechanisms of fuzi against chronic heart failure in animals: A systematic review and meta-analysis
Source: Front Pharmacol. 2022 Sep 14;13:954253. doi: 10.3389/fphar.2022.954253 (PMC9515783; doi:10.3389/fphar.2022.954253)
Supplement: Supplementary file 12 [file Table10.pdf]

Table 10 Subgroup analysis according to −dp/dtmax

Variables	Participants(n)	MD [95% CI]	P value (Significance tests)
MODEL of CHF			
drug(DOX)	169	-1.9e+03 [-2.8e+03, -981.596]	0.000
surgery(AAC)	86	-381.768 [-1.1e+03, 324.556]	0.289
Duration			
<21days	138	-2.4e+03 [-3.2e+03, -1.7e+03]	0.000
≥21days	117	-511.883 [-1.2e+03, 134.367]	0.121
